# Quantum Polar Duality and the Symplectic Camel: A New Geometric Approach to Quantization

**DOI:** 10.1007/s10701-021-00465-6

**Published:** 2021-05-21

**Authors:** Maurice A. de Gosson

**Affiliations:** University of Vienna Faculty of Mathematics (NuHAG), Wien, Austria

**Keywords:** Quantum polar duality, Covariance ellipsoid, Uncertainty principle, Pauli problem, Symplectic camel, Symplectic capacity

## Abstract

We define and study the notion of quantum polarity, which is a kind of geometric Fourier transform between sets of positions and sets of momenta. Extending previous work of ours, we show that the orthogonal projections of the covariance ellipsoid of a quantum state on the configuration and momentum spaces form what we call a dual quantum pair. We thereafter show that quantum polarity allows solving the Pauli reconstruction problem for Gaussian wavefunctions. The notion of quantum polarity exhibits a strong interplay between the uncertainty principle and symplectic and convex geometry and our approach could therefore pave the way for a geometric and topological version of quantum indeterminacy. We relate our results to the Blaschke–Santaló inequality and to the Mahler conjecture. We also discuss the Hardy uncertainty principle and the less-known Donoho–Stark principle from the point of view of quantum polarity.

## Introduction

The notion of duality is omnipresent in science and philosophy, and in human thinking [38]. Duality in science is usually implemented using a transformation which serves as a dictionary for translating between two different representations of an object. In quantum mechanics this role is played by the Fourier transform which allows one to switch from the position representation to the momentum representation. In this article we introduce a new kind of duality in quantum mechanics, having its roots in convex geometry. While the Fourier transform turns a function in *x*-space into a function in *p*-space our duality turns a set of positions into a set of momenta: it is thus a kind of proto-Fourier transform operating between sets, and not functions. The definition of this duality is actually very simple and it is therefore somewhat surprising that it hasn’t been noticed or used earlier in the literature. It goes as follows: let *X* be a convex body in configuration space ${\mathbb {R}}_{x}^{n}$; we assume that *X* contains the origin. This set may be, for instance, the convex closure of a cloud of position measurements performed on some physical system located near the origin. To *X* we associate its *polar dual* $X^{\hbar }$. It is, by definition, the set of all points $p=(p_{1},\ldots ,p_{n})$ in momentum space ${\mathbb {R}}_{p}^{n}$ such that we have

$$\begin{aligned} p_{1}x_{1}+\cdots +p_{n}x_{n}\le \hbar \end{aligned}$$

for all values $x=(x_{1},\ldots ,x_{n})$ in *X*. It turns out that the correspondence $X\longleftrightarrow X^{\hbar }$ is in a sense a geometric variant of the correspondence $\psi \longleftrightarrow {\widehat{\psi }}$ between a wavefunction $\psi$ and its Fourier transform and thus contains the uncertainty principle in disguise. Somewhat oversimplifying, we could say that:

*A quantum system localized in the position representation in a set* *X* *cannot be localized in the momentum representation in a set smaller than its polar dual* $X^{\hbar }$.

The following simple example illustrates this interpretation. Consider a pure quantum state $|\psi \rangle$ on the *x* axis; we assume for simplicity that the state is centered at $\langle x\rangle =\langle p\rangle =0$. That state has a covariance matrix

1 $$\begin{aligned} \Sigma = \begin{pmatrix} \sigma _{xx} &{}\quad \sigma _{xp}\\ \sigma _{px} &{}\quad \sigma _{pp} \end{pmatrix} { \ },{ \ }\sigma _{xp}=\sigma _{px} \end{aligned}$$

where $\sigma _{xx}=\langle {\widehat{x}}^{2}\rangle$, $\sigma _{pp}=\langle {\widehat{p}}^{2}\rangle$, and $\sigma _{xp}=\frac{1}{2}\langle {\widehat{x}} {\widehat{p}}+{\widehat{p}}{\widehat{x}}\rangle$. The determinant of $\Sigma$ is $D=\sigma _{xx}\sigma _{pp}-\sigma _{xp}^{2}$ and in view of the uncertainty principle in its strong form (the Robertson–Schrödinger inequality) we must have $D\ge \tfrac{1}{4}\hbar ^{2}$. We associate with $\Sigma$ the covariance ellipse $\Omega$: it is the set of all points $z=(x,p)$ in the phase plane such that $\frac{1}{2}\Sigma ^{-1}z\cdot z\le 1$; in the coordinates *x*, *p*

2 $$\begin{aligned} \Omega :~\dfrac{\sigma _{pp}}{2D}x^{2}-\frac{\sigma _{xp}}{D}px+\dfrac{\sigma _{xx}}{2D}p^{2}\le 1~. \end{aligned}$$

The orthogonal projections $\Omega _{X}$ and $\Omega _{P}$ of $\Omega$ on the *x* and *p* axes are the intervals

3 $$\begin{aligned} \Omega _{X}=[-\sqrt{2\sigma _{xx}},\sqrt{2\sigma _{xx}}]{\ {\ , \ } }\Omega _{P}=[-\sqrt{2\sigma _{pp}},\sqrt{2\sigma _{pp}}\dot{]}~. \end{aligned}$$

Let $\Omega _{X}^{\hbar }$ be the polar dual of $\Omega _{X}$: it is the set of all numbers *p* such that

$$\begin{aligned} px\le \hbar { \ \textit{for} }-\sqrt{2\sigma _{xx}}\le x\le \sqrt{2\sigma _{xx}} \end{aligned}$$

and is thus the interval

$$\begin{aligned} \Omega _{X}^{\hbar }=[-\hbar /\sqrt{2\sigma _{xx}},\hbar /\sqrt{2\sigma _{xx}}]~. \end{aligned}$$

We make the following crucial observation: since $\sigma _{xx}\sigma _{pp} \ge \frac{1}{2}\hbar$ we have the inclusion

4 $$\begin{aligned} \Omega _{X}^{\hbar }\subset \Omega _{P} \end{aligned}$$

and this inclusion reduces to the equality $\Omega _{X}^{\hbar }=\Omega _{P}$ if and only if the Heisenberg inequality is saturated (*i.e.* $\sigma _{xx}\sigma _{pp}=\frac{1}{4}\hbar ^{2}$); this corresponds to the case where the state $|\psi \rangle$ is the minimum uncertainty Gaussian

$$\begin{aligned} \psi _{0}(x)=\left( \tfrac{1}{2\pi \sigma _{xx}}\right) ^{1/4}e^{-\frac{x^{2} }{4\sigma _{xx}}}~. \end{aligned}$$

This example suggests that the uncertainty principle (UP) can be expressed using polar duality, which is a tool from convex geometry. In fact, we will see that it allows for a more general expression of the UP, of which the traditional one, using variances and covariances is a particular case.

In the present work we will extend this discussion to states with arbitrary numbers of freedoms; the approach we outline is both simple and subtle and is closely related to open problems in geometry (the Mahler conjecture, Sect. 5.1). We will see that the notion of quantum polarity is not only important from a foundational point of view, but also very fruitful for solving “practical” problems. For instance we will show that it plays an essential role for the understanding and resolution of Pauli’s reconstruction problem [43] (Theorems 10 and 12). Historically, the problem goes back to the famous question Pauli asked in [43], whether the probability densities $|\psi (x)|^{2}$ and $|{\widehat{\psi }}(p)|^{2}$ uniquely determine the wavefunction $\psi (x)$.

On a more conceptual level, it turns out that the properties of quantum polar duality can be reformulated in terms of a notion from symplectic topology, the “principle of the symplectic camel”. In [11] we already suggested that this deep and surprising principle might well be the “tip of an iceberg”. Here we go a few steps further; our analysis in [11] was based on the usual formulation of the uncertainty principle in terms of (co-)variances of quantum observables, which has a long story following the work of Heisenberg, Schrödinger, Weyl, Kennard, Robertson and many others. However, as pointed our by several authors, standard deviations only give adequate measurements of the spread for states that are Gaussian, or close to Gaussian states (Hilgevoord and Uffink [30, 31], Sharma et al. [48]; also see Butterfield’s interesting analysis [7]). It seems to us that the more geometric approach outlined in the present paper helps to avoid this pitfall. Even if some of the consequences of polar duality can be stated in terms of covariance matrices and standard uncertainty principles, these appear as secondary objects: the use of quantum dual pairs liberates the UP from the traditional use of tools from classical statistics and probability theory, such as variances and covariances.

In previous work [13] the expression of the UP in terms of covariance matrices and ellipsoids led us to define the notion of “quantum blob”, the smallest unit of phase space allowed by the UP in its traditional Robertson–Schrödinger form. The “philosophy” behind the introduction of quantum polar dual pairs is the following: instead of talking about measurements and their statistical properties (which are always related to some underlying quasi-probability distribution, we proceed purely geometrically by associating to every convex body *X* in position space its quantum polar dual $X^{\hbar }$; the product $X\times X^{\hbar }$ then forms a kind of phase space “quantum cell”, always containing a quantum blob, but the definition of $X\times X^{\hbar }$, as opposed to that of quantum blobs, is independent of any particular given state. In a sense, this new kind of “coarse-graining” may be more physical since the primary object, *X*, is a subset of the physical space ${\mathbb {R}}_{x}^{n}$ which is “Fourier transformed” by polar duality into a subset of momentum space ${\mathbb {R}}_{p}^{n}$ as in traditional quantum mechanics, where one associates to a wavefunction its Fourier transform. But there is no wavefunction here!

### Notation and Terminology

We denote by ${\mathbb {R}}_{z}^{2n}\equiv {\mathbb {R}}_{x}^{n}\times {\mathbb {R}} _{p}^{n}$ the phase system of a system with *n* degrees of freedom; it comes equipped with the standard symplectic form $\omega (z,z^{\prime })=Jz\cdot z^{\prime }=(z^{\prime })^{T}Jz$ where

$$\begin{aligned} J= \begin{pmatrix} 0_{n\times n} &{}\quad I_{n\times n}\\ -I_{n\times n} &{}\quad 0_{n\times n} \end{pmatrix} \end{aligned}$$

is the standard symplectic matrix. The symplectic group associated with $\omega$ is denoted by ${\text {Sp}}(n)$; it consists of all linear automorphisms *S* of phase space such that $\omega (Sz,Sz^{\prime } )=\omega (z,z^{\prime })$ for all $z,z^{\prime }$ in ${\mathbb {R}}_{z}^{2n}$; equivalently $S^{T}JS=SJS^{T}=J$. The metaplectic group ${\text {Mp}} (n)$ is a group of unitary operators on $L^{2}({\mathbb {R}}^{n})$ which is a double covering of ${\text {Sp}}(n)$: to every $S\in {\text {Sp}} (n)$ correspond two operators $\pm {\widehat{S}}\in {\text {Mp}}(n)$. We denote by ${\text {Symp}}(n)$ the group of all canonical transformations (= symplectomorphisms) of $({\mathbb {R}}_{z}^{2n},\omega )$: $f\in {\text {Symp}}(n)$ if and only if *f* is a diffeomorphism of ${\mathbb {R}}_{z}^{2n}$ and $f^{*}\omega =\omega$; equivalently *f* is bijective, infinitely differentiable and with infinitely differentiable inverse, and the Jacobian matrix *Df*(*z*) is symplectic for every *z*.

We will use the Löwner partial ordering of matrices [6] :$A\ge B$ (*resp*. $A>B$) means that $A-B$ is positive semidefinite (*resp*. positive definite). When writing $A>0$ it is always understood that $A=A^{T}$ ($A^{T}$ the transpose of *A*).

The *n*-dimensional Fourier transform ${\widehat{\psi }}=F\psi$ of $\psi \in L^{2}({\mathbb {R}}^{n})$ is defined for $\psi \in L^{1}({\mathbb {R}}^{n})\cap L^{2}({\mathbb {R}}^{n})$ by

5 $$\begin{aligned} {\widehat{\psi }}(p)=\left( \tfrac{1}{2\pi \hbar }\right) ^{n/2}\int e^{-\frac{i}{\hbar }px}\psi (x)d^{n}x~. \end{aligned}$$

## Background Material

We begin by recalling the main properties of density matrices; for a detailed rigorous review see [17]. We thereafter introduce the basic notions from harmonic analysis that we will need.

### Density Matrices and Their Covariance Ellipsoids

We recall some material about the density matrix formalism following our presentation in [17].

#### Density Matrices and Their Wigner Distributions

Mixed quantum states will be as usual identified with their density matrices which are convex sums of projection operators on rays ${\mathbb {C}}\psi _{j}$

$$\begin{aligned} {\widehat{\rho }}=\sum _{j}\lambda _{j}|\psi _{j}\rangle \langle \psi _{j}|~. \end{aligned}$$

A quantum state ${\widehat{\rho }}$ on $L^{2}({\mathbb {R}}_{x}^{n})$ is a positive semidefinite ${\widehat{\rho }}\ge 0$ (and hence self-adjoint) operator on $L^{2}({\mathbb {R}}_{x}^{n})$ with trace ${\text {Tr}}({\widehat{\rho }} )=1$. Such an operator is always compact and hence bounded. By definition the Wigner distribution of the state ${\widehat{\rho }}$ is the function $W_{{\widehat{\rho }}}\in L^{2}({\mathbb {R}}_{z}^{2n})$ defined by

6 $$\begin{aligned} W_{{\widehat{\rho }}}=\sum _{j}\lambda _{j}W\psi _{j} \end{aligned}$$

where $W\psi _{j}$ is the usual Wigner transform of $\psi _{j}$: for $\psi \in L^{2}({\mathbb {R}}^{n})$

7 $$\begin{aligned} W\psi (x,p)=\left( \tfrac{1}{2\pi \hbar }\right) ^{n}\int e^{-\frac{i}{\hbar }py}\psi \left( x+\tfrac{1}{2}y\right) \psi ^{*}\left( x-\tfrac{1}{2}y\right) d^{n}y~. \end{aligned}$$

The Wigner distribution of ${\widehat{\rho }}$ is conventionally written in bra-ket notation

8 $$\begin{aligned} W_{{\widehat{\rho }}}(z)=\left( \tfrac{1}{2\pi \hbar }\right) ^{n}\int e^{-\frac{i}{\hbar }py}\left\langle x+\tfrac{1}{2}y\right| {\widehat{\rho }}\left| x-\tfrac{1}{2}y\right\rangle d^{n}y \end{aligned}$$

but we will not use this notation.

#### The Covariance Matrix and Ellipsoid

Assuming that $W\psi _{j}\in L^{1}({\mathbb {R}}^{n})\cap L^{2}({\mathbb {R}}^{n})$ is $L^{2}$ normalized for each *j* the Wigner distribution $W_{{\widehat{\rho }} }(z)$ plays the role of a quasi probability distribution on phase space; this is illustrated by the marginal properties

9 $$\begin{aligned} \int W_{{\widehat{\rho }}}(z)d^{n}p&=\sum _{j}\lambda _{j}|\psi _{j} (x)|^{2} \end{aligned}$$

10 $$\begin{aligned} \int W_{{\widehat{\rho }}}(z)d^{n}x&=\sum _{j}\lambda _{j}|\widehat{\psi _{j} }(p)|^{2}~. \end{aligned}$$

Assuming in addition that the $W\psi _{j}$ decrease sufficiently fast at infinity to ensure the existence of first and second moments, one defines the covariance matrix of ${\widehat{\rho }}$ by

11 $$\begin{aligned} \Sigma = \begin{pmatrix} \Sigma _{XX} &{}\quad \Sigma _{XP}\\ \Sigma _{PX} &{}\quad \Sigma _{PP} \end{pmatrix} { \ },\quad { \ }\Sigma _{PX}=\Sigma _{XP}^{T} \end{aligned}$$

with $\Sigma _{XX}=(\sigma _{x_{j}x_{k}})_{1\le j,k\le n}$, $\Sigma _{PP}=(\sigma _{p_{j}p_{k}})_{1\le j,k\le n}$, and $\Sigma _{XP} =(\sigma _{x_{j}p_{k}})_{1\le j,k\le n}$. Assuming for notational simplicity that the first moments vanish

12 $$\begin{aligned} \int x_{j}W_{{\widehat{\rho }}}(z)d^{2n}z=\int p_{j}W_{{\widehat{\rho }}} (z)d^{2n}z=0 \end{aligned}$$

the covariances $\sigma _{x_{j}x_{k}}$ are defined by the integrals

13 $$\begin{aligned} \sigma _{x_{j}x_{k}}=\int x_{j}x_{k}W_{{\widehat{\rho }}}(z)d^{2n}z \end{aligned}$$

and similar formulas for $\sigma _{x_{j},p_{k}}$ and $\sigma _{p_{j},p_{k}}$. In more compact form,

14 $$\begin{aligned} \Sigma =\int zz^{T}W_{{\widehat{\rho }}}(z)d^{2n}z \end{aligned}$$

where *z*, *x* and *p* are viewed as column vectors. A crucial fact [10, 23, 40, 49] is that the covariance matrix $\Sigma$ satisfies the “quantum condition”

15 $$\begin{aligned} \Sigma +\frac{i\hbar }{2}J\ge 0~. \end{aligned}$$

This condition implies in particular that $\Sigma >0$ [40] (hence $\Sigma$ is invertible). Condition (15) is necessary (but not sufficient except in the Gaussian case [20]) for the positivity condition ${\widehat{\rho }}\ge 0$ to hold [11, 21], and implies the Robertson–Schrödinger uncertainty principle (RSUP)

16 $$\begin{aligned} \sigma _{x_{j}x_{j}}\sigma _{p_{j}p_{j}}\ge \sigma _{x_{j}p_{j}}^{2}+\tfrac{1}{4}\hbar ^{2} \end{aligned}$$

for $1\le j\le n$. To see this it suffices to use Sylvester’s criterion for the leading principal minors of a positive matrix, which implies that we must have

$$\begin{aligned} \begin{vmatrix} \sigma _{x_{j}x_{j}}&\quad \sigma _{x_{j}p_{j}}+\frac{i\hbar }{2}\\ \sigma _{x_{j}p_{j}}-\frac{i\hbar }{2}&\quad \sigma _{p_{j}p_{j}} \end{vmatrix} >0 \end{aligned}$$

which is equivalent to (16). Consider now the covariance ellipsoid of ${\widehat{\rho }}$; it is the phase space ellipsoid

17 $$\begin{aligned} \Omega =\left\{ z:\frac{1}{2}\Sigma ^{-1}z\cdot z\le 1\right\} \end{aligned}$$

where we are using the notation $\Sigma ^{-1}z\cdot z=z^{T}\Sigma ^{-1}z$. We have proven in [11] that the conditions (15), (16) are equivalent to the following statement:

18 $$\begin{aligned} {\textit{There \,exists} }\,S\in {\text {Sp}}(n)\,{ \textit{such\, that\,} }S(\mathcal {B}^{2n}(\sqrt{\hbar }))\subset \Omega \end{aligned}$$

where $\mathcal {B}^{2n}(\sqrt{\hbar })$ is the phase space ball with radius $\sqrt{\hbar }$ centered at the origin; this condition can in turn be rephrased in terms of the topological notion of symplectic capacity (see Sect. 2.3). In [13] (also see [9]) we have called the minimum uncertainty ellipsoids $S(\mathcal {B}^{2n}(\sqrt{\hbar }))$ “quantum blobs” hence the quantum condition (15) amounts to saying that

19 $$\begin{aligned} {\textit{The \,covariance \,ellipsoid\,} }\,\Omega \,{\textit{\,contains\, a\, quantum\, blob.}} \end{aligned}$$

### Symplectic and Metaplectic Covariance Properties

We are using Weyl’s quantization scheme (Weyl correspondence) in this paper. One of its hallmarks is its symplectic/metaplectic covariance properties.

#### Symplectic Covariance

Density matrices and their Wigner distribution enjoy a covariance property with respect to (linear) symplectic transformations. The idea is that if we make a symplectic change of coordinates, the effect is that the corresponding density operator will be changed by conjugation with any one of the two associated metaplectic operators. More precisely, let us write $W_{{\widehat{\rho }}}\leftrightarrows {\widehat{\rho }}$ the one-to-one correspondence between Wigner distributions and the corresponding density matrices. Then [10, 12, 17, 34], for every $S\in {\text {Sp}}(n)$

$$\begin{aligned} W_{{\widehat{\rho }}}\circ S^{-1}\leftrightarrows {\widehat{S}}{\widehat{\rho }}{\widehat{S}}^{-1} \end{aligned}$$

where $\pm {\widehat{S}}$ $\in {\text {Mp}}(n)$ corresponds to *S*. In particular, when ${\widehat{\rho }}$ describes a pure state $|\psi \rangle$ this becomes

$$\begin{aligned} W\psi (S^{-1}z)=W({\widehat{S}}\psi )(z)~. \end{aligned}$$

These formulas are actually particular cases of the general symplectic covariance property of Weyl calculus, which plays an essential role in the study of the symmetry properties of quantization.

The symplectic covariance property allows one to describe the action of symplectic transformations on the covariance ellipsoid $\Omega$ in terms of the state ${\widehat{\rho }}$ and its Wigner distribution $W_{{\widehat{\rho }}}$. The following table summarizes these properties20

#### The Generators of ${\text {Sp}}(n)$ and ${\text {Mp}}(n)$

For practical purposes, let us describe a simple class of generators of ${\text {Mp}}(n)$. Defining, for symmetric *P* and invertible *L*,

21 $$\begin{aligned} V_{-P}= \begin{pmatrix} I_{n\times n} &{}\quad 0_{n\times n}\\ P &{}\quad I_{n\times n} \end{pmatrix} { \ },{ \ }M_{L}= \begin{pmatrix} L^{-1} &{}\quad 0_{n\times n}\\ 0_{n\times n} &{}\quad L^{T} \end{pmatrix} \end{aligned}$$

the symplectic group ${\text {Sp}}(n)$ is generated by the set of all matrices $V_{-P}$ and $M_{L}$ together with the standard symplectic matrix *J*. To these generators of ${\text {Sp}}(n)$ correspond the generators $\pm {\widehat{V}}_{-P}$, $\pm {\widehat{M}}_{L,m}$, and $\pm {\widehat{J}}$ of the metaplectic group, given by

22 $$\begin{aligned} {\widehat{V}}_{-P}\psi (x)=e^{\frac{i}{2\hbar }Px^{2}}{ \ },{ \ }{\widehat{M}}_{L,m}\psi (x)=i^{m}\sqrt{|\det L|}\psi (Lx) \end{aligned}$$

where the integer *m* corresponds to a choice of $\arg \det L$, and

23 $$\begin{aligned} {\widehat{J}}\psi (x)=i^{-n/2}{\widehat{\psi }}(x)=\left( \tfrac{1}{2\pi \hbar i}\right) ^{n/2}\int e^{-\frac{i}{\hbar }x\cdot x^{\prime }}\psi (x^{\prime })d^{n}x^{\prime }~. \end{aligned}$$

For a detailed discussion of the properties of ${\text {Mp}}(n)$ and its generators see [10, 17].

### The Symplectic Camel and Related Objects

#### Gromov’s Symplectic Non-squeezing Theorem

In 1985 the mathematician Gromov [28] proved the following remarkable and highly non-trivial result: let $Z_{j}^{2n}(r)$ be the phase space cylinder defined by $x_{j}^{2}+p_{j}^{2}\le r^{2}$ and $\mathcal {B} ^{2n}(R)$ the centered phase space ball with radius *R*. There exists a canonical transformation *f* of ${\mathbb {R}}_{z}^{2n}$ such that $f(\mathcal {B} ^{2n}(R))\subset Z_{j}^{2n}(r)$ if and only $R\le r$. This result (the symplectic non-squeezing theorem) was reformulated by Gromov and Eliashberg [26] in the following form: let *f* be a canonical transformation of ${\mathbb {R}}_{z}^{2n}$ and $\Pi _{j}$ the orthogonal projection ${\mathbb {R}} _{z}^{2n}\longrightarrow {\mathbb {R}}_{x_{j},p_{j}}^{2}$ on any plane of conjugate variables $x_{j},p_{j}$. Then

24 $$\begin{aligned} {\text {Area}}\Pi _{j}(f(\mathcal {B}^{2n}(R)))\ge \pi R^{2}~. \end{aligned}$$

Of course the second result trivially implies the first, while the converse implication follows from the fact that any planar domain of area smaller than $\pi R^{2}$ can be mapped into a disk of the same area by an area-preserving diffeomorphism. This result is called—with a slight abuse of language—the *principle of the symplectic camel*. We have used the latter in [11] to reformulate the quantum uncertainty principle (see below), using the related notion of *symplectic capacity*. This principle demonstrates that Gromov’s theorem can be viewed as a watermark of quantum mechanics in classical (Hamiltonian) mechanics; see the discussions in [19, 21]; in the latter “the imprints of the quantum world in classical mechanics” are discussed from the point of view of symplectic topology. Also see the discussion in [32] from the Hamiltonian point of view.

#### Symplectic Capacities

For a detailed discussion of the notion of symplectic capacity and its applications in physics see [21].

A (normalized) symplectic capacity on $({\mathbb {R}}_{z}^{2n},\omega )$ associates to every subset $\Omega$ of ${\mathbb {R}}_{z}^{2n}$ a number $c(\Omega )\in \mathbb {[}0,+\infty \mathbb {]}$ such that the following properties hold [24, 25]:

- *Monotonicity* If $\Omega \subset \Omega ^{\prime }$ then $c(\Omega )\le c(\Omega ^{\prime })$;
- *Conformality* For every real scalar $\lambda$ we have $c(\lambda \Omega )=\lambda ^{2}c(\Omega )$;
- *Symplectic invariance* We have $c(f(\Omega ))=c(\Omega )$ for every canonical transformation $f\in {\text {Symp}}(n)$;
- *Normalization* We have, for $1\le j\le n$,
  25 $$\begin{aligned} c(\mathcal {B}^{2n}(R))=\pi R^{2}=c(Z_{j}^{2n}(R)) \end{aligned}$$
  where $Z_{j}^{2n}(R)$ is the cylinder $\{(x,p):x_{j}^{2}+p_{j}^{2}\le R^{2}\}$.

Notice that the symplectic invariance of a symplectic capacity implies in particular that

26 $$\begin{aligned} c(S(\Omega ))=c(\Omega ) \ \text {if} \ S\in {\text {Sp}}(n)~. \end{aligned}$$

The symplectic capacities $c_{\min }$ and $c_{\max }$ are defined by

27a $$\begin{aligned} c_{\min }(\Omega )&=\sup _{f\in {\text {Symp}}(n)}\{\pi R^{2} :f(\mathcal {B}^{2n}(R))\subset \Omega \} \end{aligned}$$

27b $$\begin{aligned} c_{\max }(\Omega )&=\inf _{f\in {\text {Symp}}(n)}\{\pi R^{2} :f(\Omega )\subset Z_{j}^{2n}(R)~. \end{aligned}$$

That $c_{\min }$ and $c_{\max }$ indeed are symplectic capacities follows from Gromov’s symplectic non-squeezing theorem [28]. Some terminology: $c_{\min }$ is called the “Gromov width” while $c_{\max }$ is the “cylindrical capacity”. This is because $c_{\max }$ measures the area of the base of the smallest cylinder into which a subset of phase space can be symplectically embedded. The notation $c_{\min }$ and $c_{\max }$ is motivated by the fact that they are the smallest (*resp.* the largest) symplectic capacities: every symplectic capacity *c* on $({\mathbb {R}}_{z}^{2n},\omega )$ satisfies

28 $$\begin{aligned} c_{\min }(\Omega )\le c(\Omega )\le c_{\max }(\Omega ) \end{aligned}$$

for all $$\Omega \subset {\mathbb {R}}_{z}^{2n}$$.

It should be observed that for $n>1$ symplectic capacities are not related to the notion of volume; the symplectic capacity of a set can be finite while having infinite volume (this is the case of the cylinders $Z_{j}^{2n}(R)$). Heuristically one can view a symplectic capacity as a generalization of the notion of area, or (equivalently) of that of action. For instance, it is possible to show that a particular symplectic capacity (the Hofer–Zehnder capacity [44]) of a compact convex set $\Omega$ with smooth boundary $\partial \Omega$ is the action integral $\int _{\gamma }pdx$ calculated along the shortest periodic orbit $\gamma$ carried by $\partial \Omega$ (“Hofer–Zehnder capacity”).

#### The Symplectic Capacity of an Ellipsoid

In what follows we use the notation $Mz^{2}=Mz\cdot z=z^{T}Mz$ (*M* a square matrix); $M>0$ means that *M* is symmetric: $M=M^{T}$ and positive definite,* i.e*. $Mz^{2}>0$ for all $z\ne 0$. A remarkable property is that all symplectic capacities agree on ellipsoids: if

$$\begin{aligned} \Omega =\{z\in {\mathbb {R}}_{z}^{2n}:Mz^{2}\le R^{2}\} \end{aligned}$$

where $M>0$, then for every symplectic capacity *c* on $({\mathbb {R}}_{z} ^{2n},\omega )$ we have

29 $$\begin{aligned} c(\Omega )=\pi R^{2}/\nu _{\max } \end{aligned}$$

where $\nu _{\max }$ is the largest symplectic eigenvalue of *M*. (Recall that the symplectic eigenvalues $\nu _{1},\ldots ,\nu _{n}$ of *M* are the numbers $\nu _{j}>0$ defined by the condition “ $\pm i\nu _{j}$ is an eigenvalue of *JM*”.) This property allowed us to prove in [11] that the RSUP is equivalent to the inequality

30 $$\begin{aligned} c(\Omega )\ge \pi \hbar \end{aligned}$$

when $\Omega$ is a quantum covariance ellipsoid. From this formula the symplectic invariance of the RSUP becomes obvious since we have $c(S(\Omega ))=c(\Omega )$ for every $S\in {\text {Sp}}(n)$.

## Quantum Dual Pairs and Covariance Ellipsoids

### Quantum Polar Duality

Polar duality is a very useful mathematical tool in convex and asymptotic geometry, and in functional analysis. It has also recently been used by Kalogeropoulos [32] to discuss phase space coarse-graining.

#### Polar Duality in Convex Geometry

Let *X* be a convex body in configuration space ${\mathbb {R}}_{x}^{n}$ (a convex body in an Euclidean space is a compact convex set with non-empty interior). We assume in addition that *X* contains 0 in its interior. This is the case if, for instance, *X* is symmetric: $X=-X$.

##### Definition 1

The *polar dual* of *X* is the subset

31 $$\begin{aligned} X^{\hbar }=\{p\in {\mathbb {R}}_{p}^{n}:px\le \hbar {\textit{for all} }x\in X\} \end{aligned}$$

of the dual space ${\mathbb {R}}_{p}^{n}\equiv ({\mathbb {R}}_{x}^{n})^{*}$.

Notice that it trivially follows from the definition that $X^{\hbar }$ is convex. In the mathematical literature one usually chooses $\hbar =1$, in which case one writes $X^{o}$ for the polar dual; we have $X^{\hbar }=\hbar X^{o}$. Here is an intuitive interpretation of the polar dual: *X* being convex it is the intersection of a (possibly infinite) family of half spaces (the “supporting hyperplanes” of *X*). Therefore, the polar of *X* can be seen as the convex hull of a (possibly infinite) set of points, coming from all of the supporting hyperplanes.

The following properties of the polar dual are obvious:

32 $$\begin{aligned}&{\textit{Biduality}: }(X^{\hbar })^{\hbar }=X~; \end{aligned}$$

33 $$\begin{aligned}&{\textit{Antimonotonicity: }}X\subset Y\Longrightarrow Y^{\hbar }\subset X^{\hbar }~; \end{aligned}$$

34 $$\begin{aligned}&{\textit{Scaling}: }\det L\ne 0\Longrightarrow (LX)^{\hbar }=(L^{T} )^{-1}X^{\hbar }~. \end{aligned}$$

The “smaller” *X* is, the larger $X^{\hbar }$ is. For instance, if $X=0$ (corresponding to a perfectly localized system) then $X^{\hbar }={\mathbb {R}}_{p}^{n}$, the whole momentum space. This property, reminiscent of the uncertainty principle, and of the duality of the support of a function and that of its Fourier transform, becomes particularly visible when one studies the polar duals of ellipsoids. Here are a few useful results:

##### Lemma 2

Let $\mathcal {B}_{X}^{n}(R)$ (*resp*. $\mathcal {B}_{P}^{n}(R)$) be the ball $\{x:|x|\le R\}$ in ${\mathbb {R}}_{x}^{n}$ (*resp*. $\{p:|p|\le R\}$ in ${\mathbb {R}}_{p}^{n}$). (i) We have

35 $$\begin{aligned} \mathcal {B}_{X}^{n}(R)^{\hbar }=\mathcal {B}_{P}^{n}(\hbar /R)~. \end{aligned}$$

In particular

36 $$\begin{aligned} \mathcal {B}_{X}^{n}(\sqrt{\hbar })^{\hbar }=\mathcal {B}_{P}^{n}(\sqrt{\hbar })~. \end{aligned}$$

(ii) Let $A=A^{T}$ be an invertible $n\times n$ matrix. We have

37 $$\begin{aligned} \{x:Ax^{2}\le R^{2}\}^{\hbar }=\{p:A^{-1}p^{2}\le (\hbar /R)^{2}\} \end{aligned}$$

and hence

38 $$\begin{aligned} \{x:Ax^{2}\le \hbar \}^{\hbar }=\{p:A^{-1}p^{2}\le \hbar \}~. \end{aligned}$$

##### Proof

Let us show that $\mathcal {B}_{X}^{n}(R)^{\hbar }\subset \mathcal {B}_{P} ^{n}(\hbar /R)$. Let $p\in \mathcal {B}_{X}^{n}(R)^{\hbar }$ and set $x=(R/|p|)p$; we have $|x|=R$ and hence $px\le \hbar$, that is $R|p|\le \hbar$ and $p\in \mathcal {B}_{P}^{n}(\hbar /R)$. To prove the opposite inclusion choose $p\in \mathcal {B}_{P}^{n}(\hbar /R)$. We have $|p|\le \hbar /R$ and hence, by the Cauchy–Schwarz inequality, $px\le |x||p|\le \hbar |x|/R$, that is $px\le \hslash$ for all *x* such that $|x|\le R$; this means that $p\in \mathcal {B}_{X}^{n}(R)^{\hbar }$. *(ii)* The ellipsoid $\{x:Ax^{2}\le R^{2}\}^{\hbar }$ is the image of $\mathcal {B}_{X}^{n}(R)$ by the automorphism $A^{-1/2}$; in view of formula (37) it follows from the scaling property (34) and (35) that

$$\begin{aligned} \{x:Ax^{2}\le R^{2}\}^{\hbar }=A^{1/2}\mathcal {B}_{X}^{n}(R)^{\hslash } =A^{1/2}\mathcal {B}_{P}^{n}(\hbar /R) \end{aligned}$$

which is equivalent to (37). $\square$

So far we have assumed that the convex body *X* contains the origin 0 in its interior. The definitions and results listed above extend without difficulty to the general case by picking an arbitrary $x_{0}\in X$ and replacing *X* with $X_{0}=-x_{0}+X$.

#### Quantum Dual Pairs

The following definition will be motivated by Theorem 10 below:

##### Definition 3

A pair $$(X,P)\ $$of symmetric convex bodies $X\subset {\mathbb {R}}_{x}^{n}$ and $P\subset {\mathbb {R}}_{p}^{n}$ is called a “quantum dual pair” (or, for short, “dual pair”) if we have $X^{\hbar }\subset P$ or, equivalently, $P^{\hbar }\subset X$. When equality occurs, that is if $X^{\hbar }=P$ we say that the dual pair (*X*, *P*) is saturated.

##### Remark 4

We want to make the reader aware that we are committing a slight abuse of notation and terminology here, but this abuse helps us to avoid cluttering notation and making statements unnecessarily complicated. Rigorously speaking, the set *X* is a subset the configuration space ${\mathbb {R}}_{x}^{n}$ of a system, while *P* is a subset of the momentum space ${\mathbb {R}}_{p}^{n}$ of that system, which is algebraically and topologically the dual of ${\mathbb {R}}_{x}^{n}$. This amounts to identify the phase space of the system with the cotangent bundle $T^{*}{\mathbb {R}}_{x}^{n}={\mathbb {R}}_{x}^{n} \times ({\mathbb {R}}_{x}^{n})^{*}$. However, since we will be working in “flat” configuration space we are identifying ${\mathbb {R}}_{p}^{n}$ with a copy of ${\mathbb {R}}_{x}^{n}$ and the phase space with the product ${\mathbb {R}}_{x}^{n}\times {\mathbb {R}}_{p}^{n} \equiv {\mathbb {R}}_{x,p}^{2n}\equiv {\mathbb {R}}_{z}^{2n}$.

Here are two elementary but important properties of quantum pairs:

39 $$\begin{aligned}&{\textit{Let\,} }(X,\,P)\,{\textit{be \,a \,quantum \,dual\, pair\, and\,} }\,Y,Q\,{\textit{\,be\, symmetric}\, \textit{convex }}\nonumber \\&\quad {\textit{bodies\, such \,that }}\,X\subset Y\,\text { and }\,P\subset Q\,{\textit{. Then} }\,(Y,Q){\textit{is\, also \,a\, quantum\, dual\, pair.} } \end{aligned}$$

This follows from the antimonotonicity of the passage to the dual where we have the chain of inclusions $Y^{\hbar }\subset X^{\hbar }\subset P\subset Q$;

40 $$\begin{aligned}&{\textit{Two ellipsoids\,} }X=\{x:Ax^{2}\le \hbar \}{\textit{and } }P=\{p:Bp^{2}\le \hbar \} \nonumber \\&\quad {\textit{form a quantum dual pair if and only if }}AB\le I_{n\times n}\nonumber \\&\quad {\textit{and} \textit{we have the equality}.}X^{\hbar }\,\,=P\,{\textit{\,if and only if\,} }AB=I_{n\times n}~. \end{aligned}$$

This follows from the slightly more general statement: if $X=\{x:Ax^{2}\le R^{2}\}$ and $P=\{p:Bp^{2}\le R^{\prime 2}\}$ then (*X*, *P*) is a quantum dual pair if and only if $AB\le (\hbar ^{-1}R^{\prime }R)^{2}$. In view of the duality formula (37) we have

$$\begin{aligned} X^{\hbar }=\{p:A^{-1}p^{2}\le (\hbar /R)^{2}\} \end{aligned}$$

and we thus have $X^{\hbar }\subset P$ if and only if $R^{2}\hbar ^{-2} A^{-1}x^{2}\le 1$ implies $(R^{\prime })^{-2}Bx^{2}\le 1$. But this condition is in turn equivalent to $R^{2}\hbar ^{-2}A^{-1}\ge (R^{\prime })^{-2}B$, that is $AB\le (\hbar ^{-1}R^{\prime }R)^{2}$. We have used here the following property of the Löwner ordering: if *K* and *L* are positive definite symmetric matrices such that $K^{-1}\ge L$ then $KL\le I_{n\times n}$ (and conversely): $K^{-1}\ge L$ is equivalent to $K^{1/2}LK^{1/2}\le I_{n\times n}$ and $K^{1/2}LK^{1/2}$ and *KL* have the same eigenvalues.

#### Polar Duality and Lagrangian Planes

We have defined polar duality in terms of the subspaces ${\mathbb {R}}_{x} ^{n}\equiv {\mathbb {R}}_{x}^{n}\times 0$ and ${\mathbb {R}}_{p}^{n}\equiv 0\times {\mathbb {R}}_{p}^{n}$ of the phase space ${\mathbb {R}}_{z}^{2n}$. These subspaces have the property that the symplectic form $\omega$ vanishes identically on them: $\omega (x,0;x^{\prime },0)=0$ and $\omega (0,p;0.p^{\prime })=0$ for all $x^{\prime }$ and $p^{\prime }$. Any *n*-dimensional subspace $\ell$ of ${\mathbb {R}}_{z}^{2n}$ on which $\omega$ is identically zero is called a *Lagrangian plane *in the symplectic literature [10]. The set of all Lagrangian planes in $({\mathbb {R}}_{z}^{2n},\omega )$ is denoted by ${\text {Lag}}(n)$ (or sometimes $\Lambda (n)$) and is called the *Lagrangian Grassmannian* of$({\mathbb {R}}_{z}^{2n},\omega )$. One can show that ${\text {Lag}}(n)$ can be identified with the homogeneous space *U*(*n*)/*O*(*n*) and equipped with its natural topology. Since the image of a Lagrangian plane by a symplectic transformation obviously also is a Lagrangian plane, it follows that we have a continuous and transitive action

$$\begin{aligned} {\text {Sp}}(n)\times {\text {Lag}}(n)\longrightarrow {\text {Lag}}(n)~. \end{aligned}$$

It turns out that we can define the notion of polarity for any pair $(\ell ,\ell ^{\prime })$ of transversal Lagrangian planes, that is, $\ell \cap \ell ^{\prime }=0$. To see this we begin by making the following remark which relates the notion of polarity to the symplectic structure: let *X* be, as before, a convex body in ${\mathbb {R}}_{x}^{n}\equiv {\mathbb {R}}_{x}^{n} \times 0$ containing the origin. We observe that the polar dual $X^{\hbar }$ is the subset of ${\mathbb {R}}_{p}^{n}\equiv 0\times {\mathbb {R}}_{p}^{n}$ defined by the condition

$$\begin{aligned} z\in X^{\hbar }\Longleftrightarrow \omega (z,z^{\prime })\le \hbar \text { for all }z^{\prime }\in X~\text {.} \end{aligned}$$

Indeed, since $z=(0,p)$ and $z^{\prime }=(x^{\prime },0)$ for some *p* and $x^{\prime }$ we have $\omega (z,z^{\prime })=\omega ((0,p;x^{\prime },0)=px^{\prime }$whence $z\in X^{\hbar }$ means that $px^{\prime }\le \hbar$, which is the usual definition (31) of the polar dual. This observation motivates the following definition:

##### Definition 5

Let $(\ell ,\ell ^{\prime })$ be a pair of transversal Lagrangian planes, and $X_{\ell }$ a convex body containing the origin in $\ell$. The polar dual $X_{\ell ^{\prime }}^{\hbar }$ of $X_{\ell }$ is the subset of $\ell ^{\prime }$ consisting of all $z^{\prime }\in \ell ^{\prime }$ such that

41 $$\begin{aligned} \omega (z,z^{\prime })\le \hbar \ \text {for all} \ z\in X_{\ell }~. \end{aligned}$$

This definition and the discussion that precedes it show that the notion of polar dual is actually of a deep symplectic nature. It turns out that using the properties of Lagrangian planes (see [10] for a detailed study) one can give the following equivalent definition of polar duality:

##### Definition 6

Let $(\ell ,\ell ^{\prime })$ be a pair of transversal Lagrangian planes, and set $\ell _{X}={\mathbb {R}}_{x}^{n}\times 0$, $\ell _{P}=0\times {\mathbb {R}}_{p}^{n}$. Let $S\in {\text {Sp}}(n)$ be such that $\ell =S\ell _{X}$ and $\ell ^{\prime }=S\ell _{P}$. and $X_{\ell }\subset \ell$ a convex body containing the origin. The polar dual $X_{\ell ^{\prime }}^{\hbar } \subset \ell ^{\prime }$ of $X_{\ell }$ is defined by

42 $$\begin{aligned} X_{\ell ^{\prime }}^{\hbar }=S(S^{-1}X_{\ell })^{\hbar } \end{aligned}$$

where $(S^{-1}X_{\ell })^{\hbar }$ is the polar dual of $S^{-1}X_{\ell } \subset \ell _{X}$.

The key point in the proof of the equivalence of both definitions lies in the following property of the Lagrangian Grassmannian ${\text {Lag}}(n)$: the symplectic group ${\text {Sp}}(n)$ acts transitively on pairs of transverse Lagrangian planes; *i.e*. if $(\ell _{1},\ell _{1}^{\prime })$ and $(\ell _{2},\ell _{2}^{\prime })$ are pairs of elements of ${\text {Lag}}(n)$ such that $\ell _{1}\cap \ell _{1}^{\prime }=\ell _{2} \cap \ell _{2}^{\prime }=0$ then there exists exactly one $S\in {\text {Sp}} (n)$ such that $(\ell _{1},\ell _{1}^{\prime })=S(\ell _{2},\ell _{2}^{\prime })=(S\ell _{2},S\ell _{2}^{\prime })$. Specializing to the case $(\ell _{1} ,\ell _{1}^{\prime })=(\ell ,\ell ^{\prime })$ and $(\ell _{2},\ell _{2}^{\prime })=(\ell _{X},\ell _{P})$ the existence of $S\in {\text {Sp}}(n)$ in Definition 6 follows. There remains to show that conditions (41) and (42) are indeed equivalent. Let $z^{\prime }\in X_{\ell ^{\prime }}^{\hbar }$, equivalently $S^{-1}z^{\prime }=(S^{-1}X_{\ell })^{\hbar }\in \ell _{P}$ which means that $\omega (S^{-1}z,S^{-1}z^{\prime } )\le \hbar$ for all $S^{-1}z\in \ell _{X}$. Since $\omega (S^{-1}z,S^{-1} z^{\prime })=\omega (z,z^{\prime })$ we thus have $\omega (z,z^{\prime })\le \hbar$ for all $z\in X_{\ell }$ which means that $X_{\ell ^{\prime }}^{\hbar }$ is the polar dual of $X_{\ell }$ by definition (41). Reversing the argument we see that, conversely, (41) implies (42).

The properties of this generalized notion of polar duality are similar to those in the standard case. For instance, we have the biduality property

$$\begin{aligned} (X_{\ell ^{\prime }}^{\hbar })_{\ell }^{\hbar }=X_{\ell } \end{aligned}$$

which readily follows from definition (42), swapping the roles of $\ell$ and $\ell ^{\prime }$:

$$\begin{aligned} (X_{\ell ^{\prime }}^{\hbar })_{\ell }^{\hbar }&=S[S^{-1}X_{\ell ^{\prime } }^{\hbar }]^{\hbar }=S[S^{-1}S(S^{-1}X_{\ell })^{\hbar }]^{\hbar }\\&=S[(S^{-1}X_{\ell })^{\hbar }]^{\hbar }=X_{\ell }~. \end{aligned}$$

Similarly, the antimonotonicity property (33) becomes

43 $$\begin{aligned} X_{\ell }\subset Y_{\ell }\Longrightarrow Y_{\ell ^{\prime }}^{\hbar }\subset X_{\ell ^{\prime }}^{\hbar }~. \end{aligned}$$

Let us see what happens with the scaling property (34); what follows will shed more light on its meaning which was hidden because of our identification of $\ell _{X}={\mathbb {R}}_{x}^{n}\times 0$ with ${\mathbb {R}} _{x}^{n}$. Formula (34) says that for every automorphism *L* of ${\mathbb {R}}_{x}^{n}$ (*i.e.* an invertible $n\times n$ matrix) we have $(LX)^{\hbar }=(L^{T})^{-1}X^{\hbar }$. However, if we view *X* as a subset of ${\mathbb {R}}_{x}^{n}\times 0$ it must be acted upon by automorphisms of ${\mathbb {R}}_{z}^{2n}$ (*i.e.* $2n\times 2n$ matrices). Using the symplectic matrix $$M_{L^{-1}}= \begin{pmatrix} L &{}\quad 0\\ 0 &{}\quad (L^{-1})^{T} \end{pmatrix} $$ we can view *LX* as $M_{L^{-1}}X$ when $X\subset \ell _{X}={\mathbb {R}}_{x} ^{n}\times 0$. Similarly, $(L^{T})^{-1}X^{\hbar }$ can be viewed as $M_{L^{-1} }X^{\hbar }$ when $X^{\hbar }\subset \ell _{P}=0\times {\mathbb {R}}_{p}^{n}$. With this notation the scaling formula now reads

$$\begin{aligned} (M_{L^{-1}}X)^{\hbar }=M_{L^{-1}}X^{\hbar }~. \end{aligned}$$

It turns out that this is a particular case of the following general formulas: for every $S_{0}\in {\text {Sp}}(n)$ and $X_{\ell }\subset \ell$ we have

44 $$\begin{aligned} (S_{0}X_{\ell })_{S_{0}\ell ^{\prime }}^{\hbar }=S_{0}X_{\ell }^{\hbar } \end{aligned}$$

as follows from the observation that $S_{0}$ takes $\ell$ to $S_{0}\ell$ and $\ell ^{\prime }$ to $S_{0}\ell ^{\prime }$.

The notion of dual quantum pair is defined accordingly: it is a pair $(X_{\ell },P_{\ell ^{\prime }})$ with $X_{\ell }\subset \ell$ and $P_{\ell ^{\prime }}\subset \ell ^{\prime }$ such that $X_{\ell }^{\hbar }\subset P_{\ell ^{\prime }}$. Formula (44) shows that a symplectic transformation $S_{0}\in {\text {Sp}}(n)$ takes a dual quantum pair $(X_{\ell } ,P_{\ell ^{\prime }})$ into the dual quantum pair $(S_{0}X_{\ell },S_{0} P_{\ell ^{\prime }})$ since $S_{0}P_{\ell ^{\prime }}\supset S_{0}X_{\ell }^{\hbar }=(S_{0}X_{\ell })_{S_{0}\ell ^{\prime }}^{\hbar }$. This is a geometric generalization of the symplectic invariance of the Robertson–Schrödinger inequalities [?].

#### Polar Duality and Symplectic Capacity

Let us specialize the properties above to the case of ellipsoids. We first recall the following symplectic result (see [21, 12], Sect. 6.2.1, or [16], Lemma 6):

##### Lemma 7

Let *A* and *B* be two real positive definite symmetric $n\times n$ matrices. There exists an invertible real $n\times n$ matrix *L* such that

45 $$\begin{aligned} L^{T}AL=L^{-1}B(L^{T})^{-1}=\Lambda \end{aligned}$$

where $\Lambda ={\text {diag}}(\sqrt{\lambda _{1}},\ldots ,\sqrt{\lambda _{n} })$ the $\lambda _{j}>0$ being the eigenvalues of *AB*.

Observe that the eigenvalues $\lambda _{j}$ of *AB* are $>0$ since they are the same as those of $A^{1/2}BA^{1/2}$. This result may be viewed as a special case of Williamson’s symplectic diagonalization result; we can in fact rewrite (45) as

46 $$\begin{aligned} \begin{pmatrix} A &{}\quad 0\\ 0 &{}\quad B \end{pmatrix} = \begin{pmatrix} (L^{T})^{-1} &{}\quad 0\\ 0 &{}\quad L \end{pmatrix} \begin{pmatrix} \Lambda &{}\quad 0\\ 0 &{}\quad \Lambda \end{pmatrix} \begin{pmatrix} L^{-1} &{}\quad 0\\ 0 &{}\quad L^{T} \end{pmatrix} \end{aligned}$$

and note that $$S= \begin{pmatrix} L^{-1} &{}\quad 0\\ 0 &{}\quad L^{T} \end{pmatrix} $$ is symplectic and $$S^{T}= \begin{pmatrix} (L^{T})^{-1} &{}\quad 0\\ 0 &{}\quad L \end{pmatrix} $$. A very important property is that the cylindrical symplectic capacity $c_{\max }$ of a dual quantum pair can be explicitly calculated. In fact, we have the following generalization of the relation

47 $$\begin{aligned} {\text {Area}}(X\times X^{\hbar })=4\hbar \end{aligned}$$

for intervals:

##### Theorem 8

(i) Let (*X*, *P*) be an arbitrary pair of centrally symmetric convex bodies $X\subset {\mathbb {R}}_{x}^{n}$ and $P\subset {\mathbb {R}}_{p}^{n}$; we have

48 $$\begin{aligned} c_{\max }(X\times P)=4\hbar \max \{\lambda >0:\lambda X^{\hbar }\subset P\}~. \end{aligned}$$

(ii) Assume that $X=\{x:Ax^{2}\le \hbar \}$ and $P=\{p:Bp^{2}\le \hbar \}$ with *A*, *B* symmetric and positive definite, and $AB\le I_{n\times n}$. We have

49 $$\begin{aligned} c_{\max }(X\times P)=4\hbar \max \nolimits _{j}\{\lambda _{j}^{-1}\} \end{aligned}$$

the $\lambda _{j}\le 1$ being the eigenvalues of *AB* ($\lambda _{j}>0$). In particular

50 $$\begin{aligned} c_{\max }(X\times P)\ge 4\hbar \end{aligned}$$

with equality if and only if $P=X^{\hbar }$.

##### Proof

In [1] (Remark 4.2) Artstein–Avidan *et* *al.* show that (48) holds for $\hbar =1$; an elementary rescaling argument immediately yields the general case. Let now $X=\{x:Ax^{2}\le \hbar \}$ and $P=\{p:Bp^{2}\le \hbar \}$ and choose *L* such that $L^{T}AL=L^{-1} B(L^{T})^{-1}=\Lambda$ (Lemma 7). We have

$$\begin{aligned} L^{-1}(X)&=\{x: {\textstyle \sum _{j=1}^{n}} \lambda _{j}^{1/2}x_{j}^{2}\le \hbar \}=\Lambda ^{-1/4}\mathcal {B}_{X}^{n} (\sqrt{\hbar })\\ L^{T}(P)&=\{p: {\textstyle \sum _{j=1}^{n}} \lambda _{j}^{1/2}p_{j}^{2}\le \hbar \}=\Lambda ^{-1/4}\mathcal {B}_{P}^{n} (\sqrt{\hbar })~ \end{aligned}$$

and thus

$$\begin{aligned} c_{\max }(X\times P)&=c_{\max }(L^{-1}(X)\times L^{T}(P))\\&=c_{\max }(\Lambda ^{-1/4}\mathcal {B}_{X}^{n}(\sqrt{\hbar })\times \Lambda ^{-1/4}\mathcal {B}_{P}^{n}(\sqrt{\hbar })) \end{aligned}$$

where the first equality follows from the symplectic invariance formula (26). To prove (49) let us determine the largest $\lambda >0$ such that

$$\begin{aligned}&c_{\max }(\Lambda ^{-1/4}\mathcal {B}_{X}^{n}(\sqrt{\hbar })\times \Lambda ^{-1/4}\mathcal {B}_{P}^{n}(\sqrt{\hbar }))\\&\quad = \lambda (\Lambda ^{-1/4}\mathcal {B}_{X}^{n}(\sqrt{\hbar }))^{\hbar }\subset \Lambda ^{-1/4}\mathcal {B}_{P}^{n}(\sqrt{\hbar })~. \end{aligned}$$

We have $(\Lambda ^{-1/4}\mathcal {B}_{X}^{n}(\sqrt{\hbar }))^{\hbar } =\Lambda ^{1/4}\mathcal {B}_{P}^{n}(\sqrt{\hbar })$ and hence

$$\begin{aligned} \lambda \Lambda ^{1/4}\mathcal {B}_{X}^{n}(\sqrt{\hbar }))\subset \Lambda ^{-1/4}\mathcal {B}_{P}^{n}(\sqrt{\hbar }) \end{aligned}$$

or, equivalently, $\lambda \mathcal {B}_{X}^{n}(\sqrt{\hbar })\subset \Lambda ^{-1/2}\mathcal {B}_{P}^{n}(\sqrt{\hbar })$. But this means that we must have $\lambda ^{2}\ge \lambda _{j}^{-1}$ for all $j=1,\ldots ,n$. This proves formula (49). The inequality (50) follows since $AB\le I_{n\times n}$. Suppose that $P=X^{\hbar }$; then $B=A^{-1}$ so that the eigenvalues $\lambda _{j}$ are all equal to one, hence $c_{\max }(X\times X^{\hbar })=4\hbar$. If conversely $c_{\max }(X\times P)=4\hbar$ then we must have $\max \nolimits _{j}\{\lambda _{j}^{-1}\}=1$ that is again $\lambda _{j}=1$ for all *j* which means that we have $A^{1/2}BA^{1/2}=I_{n\times n}$ and hence $B=A^{-1}$, that is $P=X^{\hbar }$. $\square$

### Polar Duality by Orthogonal Projections

#### The Schur Complement

It will be convenient to introduce the matrix

51 $$\begin{aligned} M=\frac{\hbar }{2}\Sigma ^{-1} \end{aligned}$$

in which case the covariance ellipsoid takes the form

52 $$\begin{aligned} \Omega =\{z:Mz\cdot z\le \hbar \}~. \end{aligned}$$

The matrix *M* is a real positive definite symmetric $2n\times 2n$ matrix: $M=M^{T}>0$. We will write it in block-matrix form

53 $$\begin{aligned} M= \begin{pmatrix} M_{XX} &{}\quad M_{XP}\\ M_{PX} &{}\quad M_{PP} \end{pmatrix} \end{aligned}$$

where the blocks are $n\times n$ matrices. The condition $M>0$ ensures us that $M_{XX}>0$, $M_{PP}>0$, and $M_{PX}=M_{XP}^{T}$. Recall [52] the following definition: the $n\times n$ matrices

54 $$\begin{aligned} M/M_{PP}&=M_{XX}-M_{XP}M_{PP}^{-1}M_{PX} \end{aligned}$$

55 $$\begin{aligned} M/M_{XX}&=M_{PP}-M_{PX}M_{XX}^{-1}M_{XP} \end{aligned}$$

are the *Schur complements* in *M* of $M_{PP}$ and $M_{XX}$, respectively, and we have $M/M_{PP}>0$, $M/M_{XX}>0$ [52].

We assume that the uncertainty principle in its form (15) holds. This is equivalent to the existence of $S\in {\text {Sp}}(n)$ such that $S(\mathcal {B}^{2n}(\sqrt{\hbar })\subset \Omega$ (see [10, 11, 13, 21]).

Let $\Pi _{X}$ (resp. $\Pi _{P}$) be the orthogonal projection ${\mathbb {R}} _{z}^{2n}\longrightarrow {\mathbb {R}}_{x}^{n}$ (*resp*. ${\mathbb {R}} _{z}^{2n}\longrightarrow {\mathbb {R}}_{p}^{n}$) and set

56 $$\begin{aligned} \Omega _{X}=\Pi _{X}\Omega { \ },{ \ }\Omega _{P}=\Pi _{P}\Omega ~. \end{aligned}$$

##### Lemma 9

Let $\Omega =\{z:Mz\cdot z\le \hbar \}$, $M>0$. The orthogonal projections $\Omega _{X}$ and $\Omega _{P}$ of $\Omega$ are the ellipsoids

57 $$\begin{aligned} \Omega _{X}&=\{x\in {\mathbb {R}}_{x}^{n}:(M/M_{PP})x^{2}\le \hbar \} \end{aligned}$$

58 $$\begin{aligned} \Omega _{P}&=\{p\in {\mathbb {R}}_{p}^{n}:(M/M_{XX})p^{2}\le \hbar \}~. \end{aligned}$$

##### Proof

Let us set $Q(z)=Mz^{2}-\hbar$; the boundary $\partial \Omega$ of the hypersurface $Q(z)=0$ is defined by

59 $$\begin{aligned} M_{XX}x^{2}+2M_{PX}x\cdot p+M_{PP}p^{2}=\hbar ~. \end{aligned}$$

A point *x* belongs to the boundary $\partial \Omega _{X}$ of $\Omega _{X}$ if and only if the normal vector to $\partial \Omega$ at the point $z=(x,p)$ is parallel to ${\mathbb {R}}_{x}^{n}\times 0$ hence we get the constraint $\nabla _{z}Q(z)=2Mz\in {\mathbb {R}}_{x}^{n}\times 0$; this is equivalent to saying that $M_{PX}x+M_{PP}p=0$, that is to $p=-M_{PP}^{-1}M_{PX}x$. Inserting this value of *p* in the equation (59) shows that $\partial \Omega _{X}$ is the set of all *x* such that $(M/M_{PP})x^{2}=\hbar$, which yields (57). Formula (58) is proven in the same way, swapping the subscripts *X* and *P*. $\square$

#### Proof of the Projection Theorem

Let us now prove the projection theorem (we have given a proof thereof in [14] when the covariance matrix is block-diagonal).

##### Theorem 10

Assume that the covariance ellipsoid $\Omega$ satisfies the quantization condition $\Sigma +\frac{i\hbar }{2}J\ge 0$ (resp. $M^{-1}+iJ\ge 0$). Then, the orthogonal projections $\Omega _{X}=\Pi _{X}\Omega$ and $\Omega _{P}=\Pi _{P}\Omega$ of $\Omega$ on ${\mathbb {R}}_{x}^{n}$ and ${\mathbb {R}}_{p}^{n}$, respectively, form a dual quantum pair: we have $\Omega _{X}^{\hbar }\subset \Omega _{P}$.

##### Proof

In view of property (39) it suffices to prove that there exist $Y\subset \Omega _{X}$ and $Q\subset \Omega _{P}$ such that $Y^{\hbar }\subset Q$. For this purpose we recall (property (18)) that the quantum condition $\Sigma +(i\hbar /2)J\ge 0$ is equivalent to the existence of $S\in {\text {Sp}}(n)$ such that $S(\mathcal {B}^{2n}(\sqrt{\hbar }))\subset \Omega$; it is therefore sufficient to show that the projections $Y=\Pi _{X}(S(\mathcal {B}^{2n}(\sqrt{\hbar }))$ and $Q=\Pi _{P}(S(\mathcal {B} ^{2n}(\sqrt{\hbar }))$ form a quantum pair, that is $Y^{\hbar }\subset Q$. The ellipsoid $S(\mathcal {B}^{2n}(\sqrt{\hbar }))$ consists of all $z\in {\mathbb {R}}_{z}^{2n}$ such that $Rz^{2}\le \hbar$ where $R=(SS^{T})^{-1}$. Since *R* is symmetric and positive definite we can write it in block-matrix form as

$$\begin{aligned} R= \begin{pmatrix} A &{}\quad B\\ B^{T} &{}\quad D \end{pmatrix} \end{aligned}$$

with $A>0$, $D>0$, and the projections *Y* and *Q* are given by formulas (57) and (58), which read here

$$\begin{aligned} Y&=\{x:(R/D)x^{2}\le \hbar \}\\ Q&=\{p:(R/A)p^{2}\le \hbar \}~; \end{aligned}$$

we have $R/D>0$ and $R/A>0$ [52]. In view of formula (38) we have

$$\begin{aligned} Y^{\hbar }=\{p:(R/D)^{-1}p^{2}\le \hbar \} \end{aligned}$$

hence the condition $Y^{\hbar }\subset Q$ is equivalent to

60 $$\begin{aligned} (R/D)^{-1}\ge R/A~. \end{aligned}$$

Let us prove that this inequality holds. The conditions $R\in {\text {Sp}}(n)$, $R=R^{T}$ being equivalent to $RJR=J$ we have

61 $$\begin{aligned} AB^{T}=BA{ , \ }B^{T}D=DB \end{aligned}$$

62 $$\begin{aligned} AD-B^{2}=I_{n\times n}~. \end{aligned}$$

These relations imply that the Schur complements *R*/*D* and *R*/*A* are

63 $$\begin{aligned} R/D&=(AD-B^{2})D^{-1}=D^{-1} \end{aligned}$$

64 $$\begin{aligned} R/A&=A^{-1}(AD-B^{2})=A^{-1} \end{aligned}$$

and hence the inequality (60) holds if and only $D\ge A^{-1}$. This condition is in turn equivalent to $AD\ge I_{n\times n}$. In fact, the inequality $D\ge A^{-1}$ is equivalent to $A^{1/2}DA^{1/2}\ge I_{n\times n}$; now $A^{1/2}DA^{1/2}$ and *AD* have the same eigenvalues hence $AD\ge I_{n\times n}$. If conversely $AD\ge I_{n\times n}$ then $D^{1/2}AD^{1/2}\ge I_{n\times n}$ hence $A\ge D^{-1}$ that is $D\ge A^{-1}$. Now (62) implies that $AD=I_{n\times n}+B^{2}$ hence we will have $AD\ge I_{n\times n}$ if $B^{2}\ge 0$. To prove that $B^{2}\ge 0$ we note that since $AB^{T}=BA$ (first formula (61)) we have $B^{T}=A^{-1}BA$ so that *B* and $B^{T}=B^{*}$ have the same eigenvalues and these must be real. It follows that the eigenvalues of $B^{2}$ are $\ge 0$ hence $B^{2}\ge 0$ as claimed. $\square$

We will analyze the inverse problem in Sect. 4, where we investigate whether a covariance ellipsoid $\Omega$ can be reconstructed from its orthogonal projections $\Omega _{X}$ and $\Omega _{P}$ (“Pauli’s problem”).

### Quantum Polarity and Dynamics

So far we have been dealing only with time-independent processes. Let us now have a look at quantum polarity from a dynamical point of view.

#### Quadratic Hamiltonians

The following is well-known [10, 12, 34]. Let *H* be a Hamiltonian function on phase space ${\mathbb {R}}_{z}^{2n}$; we assume that *H* is a quadratic form in the position and momentum variables $x_{j},p_{k}$. Such a function can always be written as

65 $$\begin{aligned} H(z)=\frac{1}{2}H^{\prime \prime }z\cdot z \end{aligned}$$

where $H^{\prime \prime }$ (the Hessian of *H*) is a real symmetric $2n\times 2n$ matrix ; it is convenient to rewrite this as

$$\begin{aligned} H(z)=\frac{1}{2}JXz\cdot z \end{aligned}$$

where $X=-JH^{\prime \prime }$ satisfies the condition $JX+X^{T}J=0$, *i.e.* *X* is in the symplectic Lie algebra $\mathfrak {sp}(n)$ [10]. The associated Hamilton equations are ${\dot{z}}=JXz$ and its solutions are hence $z(t)=e^{tJX}z(0)$. This means that the Hamiltonian flow determined by the quadratic Hamiltonian function *H* consists of the symplectic matrices

66 $$\begin{aligned} S_{t}=e^{tJX}\in {\text {Sp}}(n)~. \end{aligned}$$

Using the path-lifting theorem from the theory of fiber bundles, or performing a direct (cumbersome) calculation one shows that the solution of the corresponding Schrödinger equation

$$\begin{aligned} i\hbar \frac{\partial \psi }{\partial t}={\widehat{H}}(x,-i\hbar \nabla _{x})\psi \end{aligned}$$

where ${\widehat{H}}={\widehat{H}}(x,-i\hbar \nabla _{x})$ is the Weyl quantization of *H*, is given by

$$\begin{aligned} \psi (x,t)={\widehat{S}}_{t}\psi (x,0) \end{aligned}$$

where the unitary operators $\widehat{S_{t}}\in {\text {Mp}}(n)$ are defined as follows: as *t* varies, the matrices $S_{t}$ describe a smooth path in ${\text {Sp}}(n)$ passing through the identity $I_{n\times n}$ at time $t=0$. To this path corresponds a unique smooth path of metaplectic operators ${\widehat{S}}_{t}$ in ${\text {Mp}}(n)$ such that ${\widehat{S}}_{0}$ is the identity operator, and this path is precisely the quantum propagator, *i.e*. ${\widehat{S}}_{t}=e^{-i{\widehat{H}}t/\hbar }$.

The discussion above extends to the case where the Hamiltonian has time-dependent coefficients,* i.e.* is of the type

$$\begin{aligned} H(z,t)=\frac{1}{2}H^{\prime \prime }(t)z\cdot z \end{aligned}$$

where $H^{\prime \prime }(t)$ depends continuously on *t*. In this case the propagator $S_{t}$ cannot in general be written in a simple explicit form, and it is advantageous to use the time-dependent flow defined by $S_{t,t^{\prime } }=S_{t}(S_{t^{\prime }})^{-1}$. The symplectic transformation $S_{t,t^{\prime } }$ takes a phase space point $z^{\prime }$ at time $t^{\prime }$ to a point *z* at time *t*. In a similar way one can consider the Schrödinger equation

$$\begin{aligned} i\hbar \frac{\partial \psi }{\partial t}={\widehat{H}}(x,-i\hbar \nabla _{x},t)\psi \end{aligned}$$

whose solution is given by

$$\begin{aligned} \psi (x,t)={\widehat{S}}_{t,t^{\prime }}\psi (x,t^{\prime }) \end{aligned}$$

where the ${\widehat{S}}_{t,t^{\prime }}\in {\text {Mp}}(n)$ are defined by lifting the time-dependent flow $S_{t,t^{\prime }}$ to the metaplectic group; see [12] for details.

#### Time-Evolution of a Dual Quantum Pair

Let us begin by studying the orthogonal projections of a phase space ellipsoid under the action of the flow $S_{t}\in {\text {Sp}}(n)$ (to simplify the notation we are limiting ourselves here to the case of a time-independent Hamiltonian; everything carries over to the time-dependent case without difficulty). Consider an ellipsoid

67 $$\begin{aligned} \Omega =\{z:Mz^{2}\le \hbar \} \end{aligned}$$

where as usual it is assumed that $M>0$. We have seen (Theorem 10) that if $\Omega$ satisfies the quantum condition $M^{-1}+iJ\ge 0$ (which we assume from now on) then the projections $\Omega _{X}$ and $\Omega _{P}$ on the coordinate Lagrangian planes $\ell _{X}={\mathbb {R}}_{x}^{n}\times 0$ and $\ell _{P}=0\times {\mathbb {R}}_{x}^{n}$ form a dual quantum pair. Let $S_{t} \in {\text {Sp}}(n)$ be the flow determined by the Hamilton equations associated with the quadratic Hamiltonian function (65). As time elapses, the ellipsoid $\Omega$ will deform into a new ellipsoid

$$\begin{aligned} \Omega _{t}=S_{t}(\Omega )=\{z:M_{t}z^{2}\le \hbar \} \end{aligned}$$

where $M_{t}=S_{-t}^{T}MS_{-t}$ (we have $S_{t}^{-1}=S_{-t}$). It is easily seen that $M_{t}$ satisfies the quantum condition $M_{t}^{-1}+iJ\ge 0$: since $S_{t}$ is symplectic we have $S_{t}JS_{t}^{T}=J$ and hence

$$\begin{aligned} M_{t}^{-1}+iJ=S_{t}MS_{t}^{T}+iJ=S_{t}(M^{-1}+iJ)S_{t}^{T}\ge 0~. \end{aligned}$$

It follows from Theorem 10 that the orthogonal projections $\Omega _{t,X}$ and $\Omega _{t,P}$ again form a dual quantum pair; in fact these projections can be calculated using formulas (57) and (58):

68 $$\begin{aligned} \Omega _{t,X}&=\{x:(M_{t}/M_{t,PP})x^{2}\le \hbar \} \end{aligned}$$

69 $$\begin{aligned} \Omega _{t,P}&=\{p:(M_{t}/M_{t,XX})p^{2}\le \hbar \} \end{aligned}$$

where $M_{t}/M_{t,PP}$ and $M_{t}/M_{t,XX}$ are the Schur complements in $M_{t}$. We will not write the explicit formulas in terms of *M* and $S_{t}$ here (they are quite complicated to work out), but rather focus on the volumes of the corresponding ellipsoids. The Schur complement satisfies the identity [52]

$$\begin{aligned} \det M_{PP}\det (M/M_{PP})=\det M_{XX}\det (M/M_{XX})=\det M \end{aligned}$$

hence we will have, since $\det M_{t}=\det M$,

$$\begin{aligned} \det (M_{t}/M_{t,PP})&=\frac{\det M}{\det M_{t,PP}} \det (M_{t}/M_{t,XX})&=\frac{\det M}{\det M_{t,XX}}~. \end{aligned}$$

It follows that

$$\begin{aligned} {\text {Vol}}\Omega _{t,X}&=\left( \frac{1}{\det M_{t,PP}}\right) ^{n}{\text {Vol}}\Omega _{X}\\ {\text {Vol}}\Omega _{t,P}&=\left( \frac{1}{\det M_{t,XX}}\right) ^{n}{\text {Vol}}\Omega _{P} \end{aligned}$$

hence the volumes of the orthogonal projections $\Omega _{t,X}$ and $\Omega _{t,P}$ are not constant in general, as opposed to the total volume ${\text {Vol}}\Omega _{t}={\text {Vol}}\Omega$ which is conserved (Liouville’s theorem). That we have a quantum-type spreading in the classical case should not be surprising; such a possibility was already pointed out by Littlejohn [34]. The reason behind this phenomenon lies in the fact that there is a one-to-one correspondence between the classical flow of a quadratic Hamiltonian and the corresponding quantum propagator, as discussed above. So, the result above is just a quantum mechanical result in disguise. In fact, suppose that

$$\begin{aligned} {\widehat{\rho }}=\sum _{j}\lambda _{j}|\psi _{j}\rangle \langle \psi _{j}| \end{aligned}$$

is a quantum state with covariance matrix ellipsoid $\Omega$ and Wigner distribution.

70 $$\begin{aligned} W_{{\widehat{\rho }}}(z)=\sum _{j}\lambda _{j}W\psi _{j}(z)~. \end{aligned}$$

The time-evolution of $W_{{\widehat{\rho }}}(z)$ (and hence of ${\widehat{\rho }}$) is given by the formula

71 $$\begin{aligned} W_{{\widehat{\rho }}}(z,t)&=\sum _{j}\lambda _{j}W({\widehat{S}}_{t}\psi _{j})(z) \end{aligned}$$

72 $$\begin{aligned}&=\sum _{j}\lambda _{j}W(\psi _{j})(S_{-t}z) \end{aligned}$$

from which it follows that the covariance ellipsoid of the evolved state ${\widehat{\rho }}_{t}$ is precisely $S_{t}(\Omega )$.

## Pauli’s Problem and Polar Duality

Wave functions do not have an immediate experimental interpretation; what may be deduced from experiments is rather the associated probability distributions $|\psi (x)|^{2}$ and $|{\widehat{\psi }}(p)|^{2}$ (or, equivalently, the Wigner transform $W\psi (x,p)$). Pauli asked in [43] the famous question whether the probability densities $|\psi (x)|^{2}$ and $|{\widehat{\psi }} (p)|^{2}$ uniquely determine the wavefunction $\psi (x)$. in Pauli’s words:

The mathematical problem as to whether, for given probability densities *W*(*p*)* and W*(*x*)*, the wavefunction *$\psi$ (...) is always uniquely determined, has still not been investigated in all its generality

We know that the answer is negative; in fact there is in general non-uniqueness of the solution, which led Corbett [8] to introduce the notion of “Pauli partners”. Mathematically speaking, the reconstruction problem we address here is that of the reconstruction of a phase space ellipsoid (subject to a quantization condition) from its orthogonal projections on the *x*- and *p*-spaces. It is a particular case of what is called quantum tomography theory; see for instance [36, 37, 39, 42].

### The Case $n=1$

We have seen that the orthogonal projections $\Omega _{X}$ and $\Omega _{P}$ of a quantum covariance matrix form a quantum dual pair. We now address the converse question: if $(\Omega _{X},\Omega _{P})$ is a dual pair of ellipsoids, is there a quantum covariance ellipsoid with orthogonal projections $\Omega _{X}$ and $\Omega _{P}$ on ${\mathbb {R}}_{x}^{n}$ and ${\mathbb {R}}_{p}^{n}$? As follows from the discussion above, such a solution, if it exists, need not be unique. Let us return to the dual pair of intervals $\Omega _{X} =[-\sqrt{2\sigma _{xx}},\sqrt{2\sigma _{xx}}]$ and $\Omega _{P}=[-\sqrt{2\sigma _{pp}},\sqrt{2\sigma _{pp}}\dot{]}$ considered in the introduction. These intervals are the orthogonal projections on the *x*- and *p*-axes, respectively, of *any* covariance ellipse $\Omega$ defined by

73 $$\begin{aligned} \dfrac{\sigma _{pp}}{2D}x^{2}-\frac{\sigma _{xp}}{D}px+\dfrac{\sigma _{xx}}{2D}p^{2}\le 1 \end{aligned}$$

where $D=\sigma _{xx}\sigma _{pp}-\sigma _{xp}^{2}\ge \frac{1}{4}\hbar ^{2}$ (formula (2)). Knowledge of the variances $\sigma _{xx}$ and $\sigma _{pp}$ does not suffice to determine uniquely $\Omega$, since we also need to know the covariance $\sigma _{xp}^{2}$. We note, however, that every ellipse (73) has area

$$\begin{aligned} {\text {Area}}(\Omega )=2\pi \sqrt{D}\ge \pi \hbar ~. \end{aligned}$$

This area condition thus excludes “thin” ellipses concentrated along a diagonal of the rectangle $\Omega _{X} \times \Omega _{P}$. Suppose that the RSUP is saturated, that is, that $D=\frac{1}{4}\hbar ^{2}$. In this case ${\text {Area}}(\Omega )=\pi \hbar$ and the relation $\sigma _{xp}^{2}=\sigma _{xx}\sigma _{pp}-\frac{1}{4}\hbar ^{2}$ determines $\sigma _{xp}$ *up to a sign*: the state ${\widehat{\rho }}$ is then either of the two pure Gaussians

74 $$\begin{aligned} \psi _{\pm }(x)=\left( \tfrac{1}{2\pi \sigma _{xx}}\right) ^{1/4}e^{-\frac{x^{2}}{4\sigma _{xx}}}e^{\pm \frac{i\sigma _{xp}}{2\hbar \sigma _{xx}}x^{2}} \end{aligned}$$

whose Fourier transforms are (up to an unimportant constant phase factor with modulus one)

75 $$\begin{aligned} {\widehat{\psi }}_{\pm }(p)=\left( \tfrac{1}{2\pi \sigma _{pp}}\right) ^{1/4}e^{-\frac{p^{2}}{4\sigma _{pp}}}e^{\mp \frac{i\sigma _{xp}}{2\hbar \sigma _{pp}}p^{2}}~, \end{aligned}$$

where $\sigma _{pp}>0$ is determined by the relation $\sigma _{xp}^{2} =\sigma _{xx}\sigma _{pp}-\frac{1}{4}\hbar ^{2}$. Both functions $\psi _{+}$ and $\psi _{-}=\psi _{+}^{*}$ and their Fourier transforms ${\widehat{\psi }}_{+}$ and ${\widehat{\psi }}_{-}$ satisfy the conditions $|\psi _{+}(x)|^{2}=|\psi _{-}(x)|^{2}$ and $|{\widehat{\psi }}_{+}(p)|^{2}=|{\widehat{\psi }}_{-}(p)|^{2}$ showing that the Pauli problem does not have a unique solution. In fact the covariance matrices determined by the states $|\psi _{\pm }\rangle$ are, respectively,

$$\begin{aligned} \Sigma _{+}= \begin{pmatrix} \sigma _{xx} &{}\quad \sigma _{xp}\\ \sigma _{px} &{}\quad \sigma _{pp} \end{pmatrix} { \ },\,\Sigma _{-}= \begin{pmatrix} \sigma _{xx} &{}\quad -\sigma _{xp}\\ -\sigma _{px} &{}\quad \sigma _{pp} \end{pmatrix} \end{aligned}$$

with $\sigma _{xp}=\sigma _{px}$ and $\sigma _{xx}\sigma _{pp}-\sigma _{xp} ^{2}=\frac{1}{4}\hbar ^{2}$; this yields two covariance ellipses $\Omega _{+}$ and $\Omega _{-}$with area $\pi \hbar$ defined by

76 $$\begin{aligned} \dfrac{\sigma _{pp}}{2D}x^{2}\mp \frac{\sigma _{xp}}{D}px+\dfrac{\sigma _{xx}}{2D}p^{2}\le 1~, \end{aligned}$$

which are symmetric by the reflections $x\rightarrow -x$ or $p\rightarrow -p$. The projections of these ellipsoids on the *x* and *p* axes are in both cases the polar dual line segments $\Omega _{X}=[-\sqrt{2\sigma _{xx}},\sqrt{2\sigma _{xx}}]$ and $\Omega _{P}=[-\sqrt{2\sigma _{pp}},\sqrt{2\sigma _{pp} }\dot{]}$.

To deal with the multidimensional case it will be convenient to use some material from the Wigner formalism.

### The Wigner and Fourier Transforms of Gaussians

We recall some well-known facts about Gaussian states and their Wigner transform. For details, proofs and generalizations see for instance [15] or [10, 34, 49]. The most general Gaussian wavefunction on ${\mathbb {R}}_{x}^{n}$ can be written

77 $$\begin{aligned} \phi _{WY}(x)=\left( \tfrac{1}{\pi \hbar }\right) ^{n/4}(\det W)^{1/4} e^{-\tfrac{1}{2\hbar }(W+iY)x^{2}} \end{aligned}$$

where *W* and *Y* are real symmetric $n\times n$ matrices with *W* positive definite. In the case $n=1$ and taking $W=\hbar /2\sigma _{xx}$ and $Y=0$ one obtains the minimum uncertainty Gaussian

$$\begin{aligned} \psi _{0}(x)=(2\pi \sigma _{xx})^{-1/4}e^{-|x|^{2}/4\sigma _{xx}}. \end{aligned}$$

The Wigner transform (7) of $\phi _{WY}$ is given by [10, 15, 34, 41]

78 $$\begin{aligned} W\phi _{WY}(z)=(\pi \hbar )^{-n}e^{-\tfrac{1}{\hbar }Gz\cdot z} \end{aligned}$$

where *G* is the symplectic symmetric positive definite matrix

79 $$\begin{aligned} G= \begin{pmatrix} W+YW^{-1}Y &{}\quad YW^{-1}\\ W^{-1}Y &{}\quad W^{-1} \end{pmatrix} ~. \end{aligned}$$

That *G* indeed is symplectic follows from the observation that $G=S^{T}S$ where

80 $$\begin{aligned} S= \begin{pmatrix} W^{1/2} &{}\quad 0\\ W^{-1/2}Y &{}\quad W^{-1/2} \end{pmatrix} \end{aligned}$$

obviously is in ${\text {Sp}}(n)$.

Using standard formulas for the calculation of Gaussian integrals (*e.g.* Lemma 241 in [12]) the Fourier transform of $\phi _{WY}$ is given by

81 $$\begin{aligned} {\widehat{\phi }}_{WY}(p)=\left( \tfrac{1}{\pi \hbar }\right) ^{n/4}(\det W)^{1/4}\det (W+iY)^{-1/2}e^{-\tfrac{1}{2\hbar }(W+iY)^{-1}p^{2}} \end{aligned}$$

where $\det (W+iY)^{-1/2}=\lambda _{1}^{-1/2}\cdot \cdot \cdot \lambda _{n}^{-1/2}$ the $\lambda _{j}^{-1/2}$ being the square roots with positive real parts of the eigenvalues $\lambda _{j}^{-1}$ of $(W+iY)^{-1}$. Using the elementary identity [51]

$$\begin{aligned} (W+iY)^{-1}=(W+YW^{-1}Y)^{-1}-iW^{-1}Y(W+YW^{-1}Y)^{-1} \end{aligned}$$

which is easily checked multiplying on the right by $W+iY$, we see that in fact

82 $$\begin{aligned} {\widehat{\phi }}_{WY}(p)=e^{i\gamma }\phi _{W^{\prime }Y^{\prime }}(p){ \ \textit{with} }\left\{ \begin{array} [c]{c} W^{\prime }=(W+YW^{-1}Y)^{-1}\\ Y^{\prime }=-W^{-1}Y(W+YW^{-1}Y)^{-1} \end{array} \right. \end{aligned}$$

where $e^{i\gamma }$ ($\gamma$ real) is a constant phase factor.

Setting $\Sigma ^{-1}=\tfrac{2}{\hbar }G$ where *G* is the symplectic matrix (79) we can rewrite its Wigner transform (88) as

83 $$\begin{aligned} W\phi _{WY}(z)=(2\pi )^{-n}\sqrt{\det \Sigma ^{-1}}e^{-\frac{1}{2}\Sigma ^{-1}z\cdot z}~. \end{aligned}$$

The inverse of *G* being readily calculated using the formula for the inverse of a symplectic matrix [10, 34] we get the explicit expression

84 $$\begin{aligned} \Sigma =\frac{\hbar }{2} \begin{pmatrix} W^{-1} &{}\quad -W^{-1}Y\\ -YW^{-1} &{}\quad W+YW^{-1}Y \end{pmatrix} ~. \end{aligned}$$

Writing $\Sigma$ in block-matrix form (11) yields the system of matrix equations

85 $$\begin{aligned} \Sigma _{XX}=\frac{\hbar }{2}W^{-1}\,,\,\Sigma _{XP}=-\frac{\hbar }{2}W^{-1}Y\,,{\ }\Sigma _{PP}=\frac{\hbar }{2}(W+YW^{-1}Y)~. \end{aligned}$$

Note that this system is overcomplete. In fact, the knowledge of partial covariance matrices allows one to determine the corresponding Gaussian state by solving the two first equalities (85) in *W* and *Y* one gets

86 $$\begin{aligned} W=\frac{\hbar }{2}\Sigma _{XX}^{-1}{ \ },{\ \ }Y=-\Sigma _{XP} \Sigma _{XX}^{-1}~; \end{aligned}$$

insertion in the third yields

87 $$\begin{aligned} \Sigma _{XP}^{2}=\Sigma _{PP}\Sigma _{XX}-\frac{\hbar ^{2}}{4}I_{n\times n}~ \end{aligned}$$

which is the matrix version of the RSUP. Notice that for given $\Sigma _{XX}$ and $\Sigma _{PP}$ the solution $\Sigma _{XP}$ is not unique. We will see (Theorem 13) that this non-uniqueness is related to the existence of “Pauli partners” in the reconstruction problem.

### The Multidimensional Case

#### Saturation of the RSUP

To generalize these constructions to the multidimensional case we begin by briefly discussing the saturation properties of the RSUP. Assume that ${\widehat{\rho }}$ is a Gaussian quantum state, that is, a state with Wigner distribution

88 $$\begin{aligned} W_{{\widehat{\rho }}}(z)=\left( \tfrac{1}{2\pi }\right) ^{n}(\det \Sigma )^{-1/2}e^{-\frac{1}{2}\Sigma ^{-1}z\cdot z} \end{aligned}$$

where $\Sigma$ satisfies the quantum condition (15). The purity of this state is

89 $$\begin{aligned} \mu ({\widehat{\rho }})={\text {Tr}}({\widehat{\rho }}^{2})=\left( \frac{\hbar }{2}\right) ^{n}(\det \Sigma )^{-1/2}~. \end{aligned}$$

In view of Williamson’s symplectic diagonalization theorem [10, 49] there exists $S\in {\text {Sp}}(n)$ such that

90 $$\begin{aligned} \Sigma =S^{T}DS\,,\,D= \begin{pmatrix} \Lambda &{}\quad 0\\ 0 &{}\quad \Lambda \end{pmatrix} {\ \textit{and}\ }\Lambda ={\text {diag}}(\nu _{1},\ldots ,\nu _{n}) \end{aligned}$$

with the $\nu _{j}>0$ being the symplectic eigenvalues of $\Sigma$ (*i.e*. the numbers $\pm i\nu _{j}$ are the eigenvalues of $J\Sigma$, that is, those of the antisymmetric matrix $\Sigma ^{1/2}J\Sigma ^{1/2}$). The quantum condition $\Sigma +\frac{i\hbar }{2}J\ge 0$ is equivalent to $\nu _{j}\ge \frac{1}{2}\hbar$ for $j=1,\ldots ,n$. The Robertson–Schrödinger inequalities are saturated, that is,

91 $$\begin{aligned} \sigma _{x_{j}x_{j}}\sigma _{p_{j}p_{j}}=\sigma _{x_{j},p_{j}}^{2}+\tfrac{1}{4}\hbar ^{2} \end{aligned}$$

for $1\le j\le n$, if and only if $\nu _{j}=\frac{1}{2}\hbar$ for all *j*, and this can only be achieved by pure Gaussian states (see [27, 47]). Formula (89), implying that ${\widehat{\rho }}$ is a pure state if and only if $\det \Sigma =(\hbar /2)^{2n}$, means, taking the factorization (90) into account, that we must have $\nu _{1}^{2} \cdot \cdot \cdot \nu _{n}^{2}=(\hbar /2)^{2n}$; since $\nu _{j}\ge \frac{1}{2} \hbar$ for all *j* we must in fact have $\nu _{1}=\cdot \cdot \cdot =\nu _{n} =\frac{1}{2}\hbar$ so that the covariance matrix has the very particular form

92 $$\begin{aligned} \Sigma =\frac{1}{2}\hbar S^{T}S{ \ },{ \ }S\in {\text {Sp}}(n) \end{aligned}$$

(this is equivalent to saying that the covariance ellipsoid is a quantum blob). The saturating states are thus those with Wigner distribution

$$\begin{aligned} W_{{\widehat{\rho }}}(z)=(\pi \hbar )^{-n}e^{-\frac{1}{\hbar }(S^{T}S)^{-1}z\cdot z} \end{aligned}$$

hence the state is the Gaussian $\phi _{WY}(x)$ defined by (77). Let

$$\begin{aligned} \phi _{0}(x)=(\pi \hbar )^{-n/4}e^{-|x|^{2}/2\hbar } \end{aligned}$$

be the standard (normalized) Gaussian state; its Wigner distribution is [10, 34, 41]

93 $$\begin{aligned} W\phi _{0}(z)=(\pi \hbar )^{-n}e^{-\frac{1}{\hbar }|z|^{2}} \end{aligned}$$

hence $W_{{\widehat{\rho }}}(z)=W\phi _{0}(S^{-1}z)$ and it follows from the symplectic covariance properties of the Wigner transform [10, 34] that the state is the Gaussian $\psi ={\widehat{S}} \phi _{0}$ where ${\widehat{S}}$ is a unitary operator (a metaplectic operator) associated with $S\in {\text {Sp}}(n)$ via the metaplectic representation of the symplectic group (see [10, 13, 34] for detailed descriptions of this method; note that in particular this shows that all pure Gaussian states can be obtained from each other using only the metaplectic group, in fact a subgroup thereof [18]). This discussion can be summarized as follows:

94 $$\begin{aligned}&{\textit{The saturation of the RSUP is equivalent to the statement}}``\Omega\, {\textit{is \,a }}\nonumber \\&\quad {\textit{quantum blob}}'', {\textit{i.e. there exists }} S\in {\text {Sp}}(n){\textit{such that }}\Omega =S(\mathcal {B} ^{2n}(\sqrt{\hbar }))~. \end{aligned}$$

Note that if $S=I_{2n\times 2n}$ then $\Omega =\mathcal {B}^{2n}(\sqrt{\hbar })$ so that the corresponding Gaussian is a minimum uncertainty state saturating the Heisenberg inequality. In fact, property (94) says that every Gaussian can be reduced to such a minimal state using a symplectic transformation [13].

#### The Reconstruction Theorem: The Saturated Case $P=X^{\hbar }$

The following intertwining lemma will allow us to reduce the study of the reconstruction problem to a “canonical” form. Recall from Section 2.2.2 that the matrices

$$\begin{aligned} M_{L}= \begin{pmatrix} L^{-1} &{}\quad 0\\ 0 &{}\quad L^{T} \end{pmatrix} { \ },{ \ }\det L\ne 0 \end{aligned}$$

are symplectic .

##### Lemma 11

Let $\Omega$ be the phase space ellipsoid defined by $Mz^{2}\le \hbar$, $M>0$. Let $\Pi _{X}$ and $\Pi _{P}$ be the orthogonal projections of ${\mathbb {R}}_{z}^{2n}$ onto ${\mathbb {R}}_{x}^{n}$ and ${\mathbb {R}}_{p}^{n}$. We have

95 $$\begin{aligned} (\Pi _{X}\times \Pi _{P})M_{L}=M_{L}(\Pi _{X}\times \Pi _{P}) \end{aligned}$$

that is

96 $$\begin{aligned} \Pi _{X}(M_{L}(\Omega ))=L^{-1}\Pi _{X}\Omega \ \text {and} \ \Pi _{P}(M_{L} (\Omega ))=L^{T}\Pi _{P}\Omega ~. \end{aligned}$$

##### Proof

The ellipsoid $M_{L}(\Omega )$ is defined by $M^{\prime }z^{2}\le \hbar$ where $M^{\prime }=(M_{L}^{T})^{-1}MM_{L}^{-1}$; a direct calculation shows that the Schur complements $M^{\prime }/M_{PP}^{\prime }$ and $M^{\prime }/M_{XX}^{\prime }$ are given by $M^{\prime }/M_{PP}^{\prime }=L^{T}(M/M_{PP})L$ and $M^{\prime }/M_{XX}^{\prime }=L^{-1}(M/M_{XX})(L^{T})^{-1}$. Formula (96) follows using (57) and (58). $\square$

Before we proceed to prove the main result of this section, let us recall ( [10], Sect. 2.1) that a block matrix

$$\begin{aligned} M= \begin{pmatrix} M_{XX} &{}\quad M_{XP}\\ M_{PX} &{}\quad M_{PP} \end{pmatrix} \end{aligned}$$

is symplectic if and only if its blocks satisfy the relations

97a $$\begin{aligned}&M_{XX}^{T}M_{PP}-M_{PX}^{T}M_{XP}=I_{n\times n} \end{aligned}$$

97b $$\begin{aligned}&M_{XX}^{T}M_{PX}{\textit{\,and\,} }M_{XP}^{T}M_{PP}\,{\textit{\,symmetric} .} \end{aligned}$$

Also recall (40) that if $X=\{x:Ax^{2}\le \hbar \}$ and $P=\{p:Bp^{2} \le \hbar \}$ with *A*, *B* symmetric and positive definite, then (*X*, *P*) is a saturated dual pair if and only if $AB=I_{n\times n}$.

##### Theorem 12

Let $X=\{x:Ax^{2}\le \hbar \}$ and $X^{\hbar }=\{p:A^{-1}p^{2} \le \hbar \}$ its quantum polar dual.

(i) The product $X\times X^{\hbar }$ contains exactly one quantum blob $\Omega =S(\mathcal {B}^{2n}(\sqrt{\hbar }))$, $S\in {\text {Sp}}(n)$, with orthogonal projections *X* and $X^{\hbar }$ on ${\mathbb {R}}_{x}^{n}$ and ${\mathbb {R}}_{p}^{n}$; that quantum blob is the ellipsoid with the largest volume inscribed in the convex set $X\times X^{\hbar }$;
(ii) $\Omega$ is the covariance ellipsoid of the pure Gaussian state
  98 $$\begin{aligned} \psi (x)=\left( \tfrac{1}{2\pi }\right) ^{n/4}(\det \Sigma _{XX})^{-1/4} e^{-\tfrac{1}{4}\Sigma _{XX}^{-1}x\cdot x} \end{aligned}$$
  where $$\Sigma _{XX}=\frac{\hbar }{2}A^{-1}$$.

##### Proof

The symplectic transformation $M_{A^{-1/2}}$ takes the dual pair $(X,X^{\hbar })$ to the dual pair $(\mathcal {B}_{X}^{n}(\sqrt{\hbar }),\mathcal {B}_{P} ^{n}(\sqrt{\hbar }))$:

99 $$\begin{aligned} (X^{\prime },X^{\prime \hbar })=M_{A^{-1/2}}(X\times X^{\hbar })=(\mathcal {B} _{X}^{n}(\sqrt{\hbar })\times \mathcal {B}_{P}^{n}(\sqrt{\hbar }))~. \end{aligned}$$

In view of Lemma 11 above, this has the effect of replacing the projections *X* and $X^{\hbar }$ with $\mathcal {B}_{X}^{n}(\sqrt{\hbar })$ and $\mathcal {B}_{P}^{n}(\sqrt{\hbar })$. By a simple symmetry argument it is seen that the John ellipsoid (which is the inscribed ellipsoid with largest volume [3, 46]) of $\mathcal {B}_{X}^{n}(\sqrt{\hbar })\times \mathcal {B}_{P}^{n}(\sqrt{\hbar })$ is the phase space ball $\mathcal {B} ^{2n}(\sqrt{\hbar })$. In view of the uniqueness of the John ellipsoid there is no other quantum blob contained in $X^{\prime }\times X^{\prime \hbar }$: assume we can find $S^{\prime }\in {\text {Sp}}(n)$ such that $S^{\prime (}\mathcal {B}^{2n}(\sqrt{\hbar }))\subset X^{\prime }\times X^{\prime \hbar }$. Since $S^{\prime }$ is volume preserving $S^{\prime }(\mathcal {B}^{2n} (\sqrt{\hbar })$ has same volume as $\mathcal {B}^{2n}(\sqrt{\hbar })$ so we must have $S^{\prime }(\mathcal {B}^{2n}(\sqrt{\hbar })=\mathcal {B}^{2n}(\sqrt{\hbar })$. The orthogonal projections of $\mathcal {B}^{2n}(\sqrt{\hbar })$ on ${\mathbb {R}}_{x}^{n}$ and ${\mathbb {R}}_{p}^{n}$ being $\mathcal {B}_{X}^{n} (\sqrt{\hbar })$ and $\mathcal {B}_{P}^{n}(\sqrt{\hbar })$, respectively, we conclude that the covariance ellipsoid we are looking for is precisely $\Omega =\mathcal {B}^{2n}(\sqrt{\hbar })$. It corresponds to the standard Gaussian $\phi _{0}(x)=(\pi \hbar )^{-n/4}e^{-|x|^{2}/2\hbar }$ whose Wigner distribution is given by

100 $$\begin{aligned} W\phi _{0}(z)=(\pi \hbar )^{-n}e^{-\frac{1}{\hbar }|z|^{2}}~. \end{aligned}$$

Returning to the original dual pair $(X,X^{\hbar })$ using (99) the covariance ellipsoid is here

$$\begin{aligned} \Omega =M_{A^{1/2}}(\mathcal {B}^{2n}(\sqrt{\hbar }))=\{z:M_{A^{-1}}z\cdot z\le \hbar \}~. \end{aligned}$$

Specializing the transformation table (20) to $S=M_{A^{-1}}$ we have the correspondences101hence the state with covariance matrix $\Omega$ is the squeezed Gaussian $\psi$ with Wigner transform

$$\begin{aligned} W\psi (z)=(\pi \hbar )^{-n}e^{-\frac{1}{\hbar }M_{A^{T}}M_{A}z\cdot z}=(\pi \hbar )^{-n}e^{-\frac{1}{\hbar }M_{A^{2}}z\cdot z}~. \end{aligned}$$

Setting $G=M_{A^{T}}M_{A}=M_{A^{2}}$ we have

$$\begin{aligned} \begin{pmatrix} W+YW^{-1}Y &{}\quad YW^{-1}\\ W^{-1}Y &{}\quad W^{-1} \end{pmatrix} = \begin{pmatrix} (A^{2})^{-1} &{}\quad 0\\ 0 &{}\quad A^{2} \end{pmatrix} \end{aligned}$$

hence $W=(A^{2})^{-1}$ and $Y=0$. In view of formulas (83) and (84) the state we are looking for is

$$\begin{aligned} \phi _{(A^{2})^{-1}0}(x)=\left( \tfrac{1}{\pi \hbar }\right) ^{n/4}(\det A)^{-1/2}e^{-\tfrac{1}{2\hbar }(A^{2})^{-1}x\cdot x}~; \end{aligned}$$

taking formula (85) into account this can be rewritten

$$\begin{aligned} \psi (x)=\phi _{(A^{2})^{-1}0}(x)=\left( \tfrac{1}{2\pi }\right) ^{n/4} (\det \Sigma _{XX})^{-1/4}e^{-\tfrac{1}{4}\Sigma _{XX}^{-1}x\cdot x} \end{aligned}$$

where $$\Sigma _{XX}=\frac{\hbar }{2}W^{-1}=\frac{\hbar }{2}A^{2}$$. $$\square $$

#### The Reconstruction Theorem in the General Case

We now consider the case $X^{\hbar }\subset P$, $X^{\hbar }\ne P$.

##### Theorem 13

Let $X=\{x:Ax^{2}\le \hbar \}$ and $P=\{p:Bp^{2}\le \hbar \}$ be two ellipsoids such that $X^{\hbar }\subset P$, $X\ne P$.

(i) The product $X\times P$ contains two quantum blobs, i.e. two (centered) ellipsoids $\Omega _{+}$ and $\Omega _{-}$ such that $\Omega _{\pm }=S_{\pm }(\mathcal {B}^{2n}(\sqrt{\hbar }))$ for some $S_{\pm }\in {\text {Sp}}(n)$ and whose orthogonal projections are *X* and *P*. These ellipsoids are the covariance ellipsoids of two pure Gaussian quantum states explicitly given by the formula
  $$\begin{aligned} \psi _{\pm }(x)=\left( \tfrac{1}{2\pi }\right) ^{n/4}(\det \Sigma _{XX} )^{-1/4}\exp \left[ -\left( \frac{1}{4}\Sigma _{XX}^{-1}\pm \frac{i}{2\hbar }\Sigma _{XP}\Sigma _{XX}^{-1}\right) x^{2}\right] \end{aligned}$$
  where $\Sigma _{XX}$ and $\Sigma _{XP}$ are the $n\times n$ matrices defined by:
  $$\begin{aligned} \Sigma _{XX}=\frac{\hbar }{2}A^{-1}\,,\,\Sigma _{XP}=\frac{\hbar }{2}(B^{-1}A^{-1}-I_{n\times n})^{1/2}~. \end{aligned}$$
(ii) Let $\Omega =\{z:\tfrac{1}{2}\Sigma ^{-1}z\cdot z\le 1\}$ be the ellipsoid with largest volume contained in $X\times P$ and having projections *X* and *P*; the quantum state with Wigner distribution
  $$\begin{aligned} W_{{\widehat{\rho }}}(z)=\left( \tfrac{1}{2\pi }\right) ^{n}(\det \Sigma )^{-1/2}e^{-\frac{1}{2}\Sigma ^{-1}z\cdot z} \end{aligned}$$
  is a mixed state with purity $\mu ({\widehat{\rho }})=\lambda _{j_{1}}^{2} \cdot \cdot \cdot \lambda _{j_{m}}^{2}$ where the $\lambda _{j_{k}}$ are the eigenvalues of *AB* that are smaller than one.

##### Proof

(i) Let us determine the quantum blobs $\Omega =S(\mathcal {B} ^{2n}(\sqrt{\hbar }))$ ($S\in {\text {Sp}}(n)$) contained in $X\times P$ and orthogonally projecting onto *X* and *P*. These will determine the functions $\psi$ we are looking for by the same procedure as in Theorem 12 via their covariance matrix $\Sigma$. Setting $M=\frac{\hbar }{2}\Sigma ^{-1}$ the condition $\Omega =S(\mathcal {B}^{2n}(\sqrt{\hbar }))$ is equivalent to $M\in {\text {Sp}}(n)$, $M>0$. The symplecticity of *M* allows us to easily invert $\Sigma$ and one finds, using (97a) and (97b),

$$\begin{aligned}\Sigma = \begin{pmatrix} \Sigma _{XX} &{}\quad \Sigma _{XP}\\ \Sigma _{PX} &{}\quad \Sigma _{PP} \end{pmatrix} =\frac{\hbar }{2} \begin{pmatrix} M_{PP} &{}\quad -M_{PX}\\ -M_{XP} &{}\quad M_{XX} \end{pmatrix} ~. \end{aligned}$$

The orthogonal projection $\Omega _{X}$ is given by the inequality $(M/M_{PP})x^{2}\le \hbar$ (Lemma 9), that is, taking again the equalities (97a) and (97b) into account and using the fact that $M_{XX}$, $M_{PP}>0$ and $M_{XP}^{T}=M_{PX}$,

$$\begin{aligned} M/M_{PP}=(M_{XX}M_{PP}-M_{XP}M_{PP}^{-1}M_{PX}M_{PP})M_{PP}^{-1}=M_{PP} ^{-1}~. \end{aligned}$$

By a similar argument we get $M/M_{XX}=M_{XX}^{-1}$ hence the equalities

102 $$\begin{aligned} A=M/M_{PP}=\frac{\hbar }{2}\Sigma _{XX}^{-1}{ \ \textit{and} \ } B=M/M_{XX}=\frac{\hbar }{2}\Sigma _{PP}^{-1}~. \end{aligned}$$

It follows that the orthogonal projections $\Omega _{X}$ and $\Omega _{P}$ are the ellipsoids

$$\begin{aligned} \Omega _{X}=\{x:\tfrac{1}{2}\Sigma _{XX}^{-1}x^{2}\le 1\}{ \ },{ \ }\Omega _{P}=\{p:\tfrac{1}{2}\Sigma _{PP}^{-1}p^{2}\le 1\}~. \end{aligned}$$

We next determine all the Gaussian states $\phi _{WY}$ having $\Omega$ as covariance matrix. As in the proof of 12, we have to solve the matrix equation

$$\begin{aligned} \begin{pmatrix} W+YW^{-1}Y &{}\quad YW^{-1}\\ W^{-1}Y &{}\quad W^{-1} \end{pmatrix} = \begin{pmatrix} M_{XX} &{}\quad M_{XP}\\ M_{PX} &{}\quad M_{PP} \end{pmatrix} ~. \end{aligned}$$

The solutions are (*cf.* formulas (86)) $W=\frac{\hbar }{2} \Sigma _{XX}^{-1}$ and $Y=-\Sigma _{XP}\Sigma _{XX}^{-1}$ corresponding to the Gaussian pure state

$$\begin{aligned} \phi _{WY}(x)= & {} \left( \tfrac{1}{2\pi }\right) ^{n/4}(\det \Sigma _{XX})^{-1/4}\\&\exp \left[ -\left( \frac{1}{4}\Sigma _{XX}^{-1}+\frac{i}{2\hbar }\Sigma _{XP}\Sigma _{XX}^{-1}\right) x\cdot x\right] \end{aligned}$$

where $\Sigma _{XP}$ is any matrix satisfying condition the matrix version (87) of the RSUP, that is

$$\begin{aligned} \Sigma _{XP}^{2}=\Sigma _{PP}\Sigma _{XX}-\frac{\hbar ^{2}}{4}I_{n\times n}~; \end{aligned}$$

Since $\Sigma _{XX}=\frac{\hbar }{2}A^{-1}$ and $\Sigma _{PP}=\frac{\hbar }{2}B^{-1}$ (formulas (102) above) this is

$$\begin{aligned} \Sigma _{XP}^{2}=\frac{\hbar ^{2}}{4}(B^{-1}A^{-1}-I_{n\times n}) \end{aligned}$$

and we are done. *(ii)* We can, as in the proof of Theorem 8, choose an invertible $n\times n$ matrix *L* such that

$$\begin{aligned} L^{T}AL=L^{-1}B(L^{T})^{-1}=\Lambda ~. \end{aligned}$$

In view of Lemma 11 above, replacing (*X*, *P*) with

103 $$\begin{aligned} X^{\prime }\times P^{\prime }=M_{L}(X\times P){ \ , \ }M_{L}= \begin{pmatrix} L^{-1} &{}\quad 0\\ 0 &{}\quad L^{T} \end{pmatrix} \end{aligned}$$

has the effect of replacing the projections $\Omega _{X}$ and $\Omega _{P}$ of an ellipsoid $\Omega$ with $L^{-1}\Omega _{X}$ and $L^{T}\Omega _{P}$. This reduces the proof to the case where *X* and *P* are replaced with

104 $$\begin{aligned} X^{\prime }=\Lambda ^{-1/4}\mathcal {B}_{X}^{n}(\sqrt{\hbar }){ \ },{\ \ }P^{\prime }=\Lambda ^{-1/4}\mathcal {B}_{P}^{n}(\sqrt{\hbar }) \end{aligned}$$

where $\Lambda ={\text {diag}}(\sqrt{\lambda _{1}},\ldots ,\sqrt{\lambda _{n} })$, the $\lambda _{j}$ being the eigenvalues of $AB\le I_{n\times n}$; the duality of *X* and *P* (and hence of $X^{\prime }$ and $P^{\prime }$) is equivalent to the conditions $0<\lambda _{j}\le 1$ for $j=1,\ldots ,n$ with at least one of the eigenvalues $\lambda _{j}$ of *AB* being $<1$ since $X^{\hbar }\ne P$ implies that $AB\le I_{n\times n}$, $AB\ne I_{n\times n}$. Explicitly:

$$\begin{aligned} X^{\prime }=\{x: {\textstyle \sum _{j=1}^{n}} \lambda _{j}^{1/2}x_{j}^{2}\le \hbar \}\,,\,P^{\prime }=\{p: {\textstyle \sum _{j=1}^{n}} \lambda _{j}^{1/2}p_{j}^{2}\le \hbar \}~. \end{aligned}$$

Now, the John ellipsoid of $X^{\prime }\times P^{\prime }$ is

$$\begin{aligned} \Omega _{\max }^{\prime }=\{(x,p): {\textstyle \sum _{j=1}^{n}} \lambda _{j}^{1/2}(x_{j}^{2}+p_{j}^{2})\le \hbar \} \end{aligned}$$

and the associated covariance matrix is

$$\begin{aligned} \Sigma _{\max }^{\prime }=\frac{\hbar }{2} \begin{pmatrix} \Lambda ^{-1/2} &{}\quad 0\\ 0 &{}\quad \Lambda ^{-1/2} \end{pmatrix} ~. \end{aligned}$$

The purity (89) of the associated Gaussian state ${\widehat{\rho }}$ is

$$\begin{aligned} \mu ({\widehat{\rho }})=\left( \frac{\hbar }{2}\right) ^{n}(\det \Sigma _{\max }^{\prime })^{-1/2}=\det \Lambda ^{2} \end{aligned}$$

and this is the square of the product of the eigenvalues of *AB* that are smaller than one. $\square$

## The Mahler Volume and Related Topics

In this section we briefly discuss some related topics where quantum polar duality can also be seen to appear, sometimes unexpectedly. Particularly interesting is the link between quantum mechanics and a well-known conjecture from convex geometry, the *Mahler conjecture*. Perhaps the Donoho–Stark uncertainty principle which is discussed thereafter might shed some new light on this difficult problem.

### The Mahler Conjecture

#### Some Known Results

Let *X* be a convex body in ${\mathbb {R}}_{x}^{n}$ (i.e. *X* is compact and has non-empty interior). We assume that *X* contains 0 in its interior. By definition, the Mahler volume [35] of *X* is the product

105 $$\begin{aligned} \upsilon (X)=|X|~|X^{\hbar }| \end{aligned}$$

where |*X*| is the usual Euclidean volume on ${\mathbb {R}}_{x}^{n}$. The Mahler volume is a dimensionless quantity because of its rescaling invariance 8see below): we have $\upsilon (\lambda X)=\upsilon (X)$ for all $\lambda >0$.

The Mahler volume is invariant under linear automorphisms of ${\mathbb {R}} _{x}^{n}$: if *L* is an automorphism of ${\mathbb {R}}_{x}^{n}$ then we have, in view of the scaling formula (34),

106 $$\begin{aligned} \upsilon (LX)=|LX|~|(L^{T})^{-1}X^{\hbar }|=|X|~|X^{\hbar }|~. \end{aligned}$$

It follows that the Mahler volume of an arbitrary ellipsoid $X=\{x:Ax^{2}\le R^{2}\}$ ($A>0$) is given by

107 $$\begin{aligned} \upsilon (X)=|\mathcal {B}^{n}(\sqrt{\hbar })|~|\mathcal {B}^{n}(\sqrt{\hbar })^{\hbar }|=\frac{(\pi \hbar )^{n}}{\Gamma (\frac{n}{2}+1)^{2}} \end{aligned}$$

and is thus the same for all ellipsoids. It turns out that the Mahler volume of ellipsoids is maximal, in the sense that we have

108 $$\begin{aligned} \upsilon (X)\le \frac{(\pi \hbar )^{n}}{\Gamma (\frac{n}{2}+1)^{2}} \end{aligned}$$

for all symmetric convex bodies, with equality occurring if and only if *X* is an ellipsoid. This result is due to Blaschke [4] for $n=2,3$ and to Santaló [45] for arbitrary *n* (see Schneider [46]).

The problem of finding a lower bound for the Mahler volume is much more difficult and a general solution is unknown. A famous conjecture, due to Mahler himself [35], says that for every symmetric convex body *X* in ${\mathbb {R}}_{x}^{n}$ we have

109 $$\begin{aligned} \upsilon (X)\ge \frac{(4\hbar )^{n}}{n!} \end{aligned}$$

with equality only when *X* is the hypercube $C=[-1,1]^{n}$. In view of the invariance property (106) this is tantamount to saying that the minimum is attained by any *n*-parallelepiped

110 $$\begin{aligned} X=[-\sqrt{2\sigma _{x_{1}x_{1}}},\sqrt{2\sigma _{x_{1}x_{1}}}]\times \cdot \cdot \cdot \times [-\sqrt{2\sigma _{x_{n}x_{n}}},\sqrt{2\sigma _{x_{n}x_{n} }}] \end{aligned}$$

which is the *n*-dimensional generalization of the interval $\Omega _{X}$ (3) of the introduction. While the conjectured inequality (109) trivially holds when $n=1$ (since $\upsilon (X)$ is just the area of the rectangle $X\times X^{\hbar }$), a proof in the general case is still lacking at the time of writing. Bourgain and Milman [5] have shown the existence, for every $n\in {\mathbb {N}}$, of a constant $C_{n}>0$ such that

111 $$\begin{aligned} |X|~|X^{\hbar }|\ge C_{n}\hbar ^{n}/n! \end{aligned}$$

and more recently Kuperberg [33] has shown that one can choose $C_{n}=(\pi /4)^{n}$, so that (111) can be rewritten

112 $$\begin{aligned} \upsilon (X)\ge \frac{(\pi \hbar )^{n}}{4^{n}n!} \end{aligned}$$

and this is the best known lower bound for the Mahler volume. Summarizing, we have the bounds

113 $$\begin{aligned} \frac{(\pi \hbar )^{n}}{4^{n}n!}\le \upsilon (X)\le \frac{(\pi \hbar )^{n}}{\Gamma (\frac{n}{2}+1)^{2}}~. \end{aligned}$$

One geometric meaning of the Mahler volume is that it captures the “roundness” of a convex body, with ellipsoids being the roundest, and cubes and octahedra being the “pointiest” [50]. It is clear that this lower bound—ideally, the conjectured bound $\upsilon (X)\ge (4\hbar )^{n}/n!$—is a form of the uncertainty principle. But what does it tell us?

#### Mahler Volume and Symplectic Capacity

We know that the notion of symplectic capacity is closely related to the uncertainty principle. There is an important inequality relating the symplectic capacity of a symmetric convex body *K* to its volume. It is the so-called *symplectic isoperimetric inequality* [1, 2] which says that

114 $$\begin{aligned} \frac{c_{\min }(K)}{c_{\min }(\mathcal {B}^{2n}(1))}\le \left( \frac{|K|}{|\mathcal {B}^{2n}(1)|}\right) ^{1/n} \end{aligned}$$

where $c_{\min }$ is the Gromov width; in other words

115 $$\begin{aligned} c_{\min }(K)\le (n!)^{1/n}|K|^{1/n}~. \end{aligned}$$

The proof of (114)–(115) is quite simple: let $\mathcal {B} ^{2n}(r)$ be the largest phase space ball that can be embedded in *K* using a canonical transformation, thus $c_{\min }(\Omega )=\pi r^{2}$. Since canonical transformations are volume preserving we have also $|K|\ge |\mathcal {B} ^{2n}(r)|$ hence the inequality $\mathcal {B}^{2n}(r)$ follows by a direct calculation. Since all symplectic capacities agree on ellipsoids the inequality (114) still holds when *K* is an ellipsoid and $c_{\min }$ is replaced with any symplectic capacity *c*. It is conjectured (“Viterbo’s conjecture”) that (114) actually holds for *all* convex bodies and *all* symplectic capacities:

116 $$\begin{aligned} c(K)\le (n!)^{1/n}|K|^{1/n} \end{aligned}$$

(see [1] for details and references). Quite surprisingly, this inequality implies the Mahler conjecture. In fact, if (116) holds, then we may choose $c=c_{\max }$ and hence, by formula (50) in Theorem 8,

$$\begin{aligned} 4\hbar =c_{\max }(X\times X^{\hbar })\le (n!)^{1/n}|X\times X^{\hbar }|^{1/n} \end{aligned}$$

that is $\upsilon (X)\ge (4\hbar )^{n}/n!$, which is the inequality (109) conjectured by Mahler.

### Hardy’s Uncertainty Principle

Let $\psi \in L^{2}({\mathbb {R}})$, $||\psi ||_{L^{2}}\ne 0$. Hardy’s uncertainty principle [29] in its original form states that we cannot have simultaneously

117 $$\begin{aligned} |\psi (x)|\le Ce^{-ax^{2}/2\hbar }{ \ },{ \ }|{\widehat{\psi }}(p)|\le Ce^{-bp^{2}/2\hbar } \end{aligned}$$

(*a*, *b*, *C* positive constants) unless $ab\le 1$ and *(i)* if $ab=1$ then $\psi (x)=\alpha e^{-ax^{2}/2\hbar }$ for some $\alpha \in {\mathbb {C}}$ and *(ii)* if $ab<1$ then $\psi$ is a finite linear combination of conveniently rescaled Hermite functions.

In the multidimensional case Hardy’s uncertainty principle can be stated as follows [21]: Let *A* and *B* be positive definite and symmetric matrices and $\psi \in L^{2}({\mathbb {R}}^{n})$, $||\psi ||_{L^{2}}\ne 0$. The Hardy inequalities

118 $$\begin{aligned} |\psi (x)|\le Ce^{-\tfrac{1}{2\hbar }Ax^{2}} \ \text {and} \ |{\widehat{\psi }}(p)|\le Ce^{-\tfrac{1}{2\hbar }Bp^{2}} \end{aligned}$$

are satisfied for some constant $C>0$ if and only if $AB\le I_{n\times n}$, that is,

119 $$\begin{aligned} {\textit{The \,eigenvalues} }\,\lambda _{1},\ldots ,\lambda _{n}\,{\textit{of} }\,AB\,{\textit{are}}\le 1 \end{aligned}$$

and we have:

(i) *If* $\lambda _{j}=1$ *for all* *j, then* $\psi (x)=\alpha e^{-\frac{1}{2\hbar }Ax^{2}}$ *for some constant *$\alpha \in {\mathbb {C}}$;
(ii) *If* $\lambda _{j}<1$ *for at least one index* *j,* *then the set of functions satisfying* (118)* is an infinite-dimensional subspace of *$L^{2}({\mathbb {R}}^{n})$*.*

In view of property (40) the conditions (119) mean that the ellipsoids

$$\begin{aligned} X_{A}=\{x:Ax^{2}\le \hbar \}{\textit{and} \ }P_{B}=\{p:Bp^{2}\le \hbar \} \end{aligned}$$

form a dual quantum pair $(X_{A},P_{B})$. If this pair is saturated (*i.e.* $P_{B}=X_{A}^{\hbar }$), then $\psi$ is a scalar multiple of the Gaussian $\phi _{AY}$. Consider now the “Hardy ellipsoid”

$$\begin{aligned} \Omega _{AB}=\{(x,p):Ax^{2}+Bp^{2}\le \hbar \} \end{aligned}$$

that is

$$\begin{aligned} \Omega _{AB}=\{z:M_{AB}z^{2}\le \hbar \}{ \ , \ }M_{AB}= \begin{pmatrix} A &{}\quad 0\\ 0 &{}\quad B \end{pmatrix} ~. \end{aligned}$$

The orthogonal projections on ${\mathbb {R}}_{x}^{n}$ and ${\mathbb {R}}_{p}^{n}$ of $\Omega _{AB}$ are precisely the ellipsoids $X_{A}$ and $P_{B}$. The symplectic eigenvalues of $M_{AB}$ are the positive numbers $\nu _{1},\ldots ,\nu _{n}$ such that $\pm i\nu _{1},\ldots ,\pm \nu _{n}$ are the solutions of the characteristic polynomial $P(t)=\det (t^{2}I_{n\times n}+AB)$ of *M*. These are the pure imaginary numbers $\pm i\sqrt{\lambda _{1}},\ldots ,\pm i\sqrt{\lambda _{n}}$ where the $\lambda _{j}>0$ are the eigenvalues of *AB*. Thus $\nu _{j}=\sqrt{\lambda _{j}}$ for $1\le j\le n$. Since we have $\lambda _{j}\le 1$ for all *j* the covariance matrix $\Sigma _{AB}=\frac{\hbar }{2}M_{AB}^{-1}$ satisfies the quantum condition $\Sigma _{AB}+\frac{i\hbar }{2}J\ge 0$; equivalently (30): $c(\Omega _{AB})\ge \pi \hbar$. If, in particular, the $\lambda _{j}$ are all equal to one we have $c(\Omega _{AB})=\pi \hbar$ and $AB=I_{n\times n}$ so that $P_{B}=X_{A}^{\hbar }$. Let us examine this case a little bit closer at the light of the reconstruction Theorems above. Assume that $\psi \in L^{2}({\mathbb {R}}^{n})$, $||\psi ||_{L^{2}}\ne 0$, and its Fourier transform satisfy

120 $$\begin{aligned} |\psi (x)|\le Ce^{-\tfrac{1}{2\hbar }Ax^{2}}\ {\textit{and} } \ |{\widehat{\psi }}(p)|\le Ce^{-\tfrac{1}{2\hbar }A^{-1}p^{2}} \end{aligned}$$

for some constant $C>0$. The ellipsoids $X_{A}$ and $P_{A^{-1}}$ are polar dual of each other hence Theorem 12 tells us that

$$\begin{aligned} \psi (x)=\left( \tfrac{1}{\pi \hbar }\right) ^{n/4}(\det A)^{1/4}e^{-\tfrac{1}{2\hbar }Ax\cdot x}~. \end{aligned}$$

The Fourier transform of $\psi$ is given (up to a constant factor with modulus one) by

$$\begin{aligned} {\widehat{\psi }}(p)=\left( \tfrac{1}{\pi \hbar }\right) ^{n/4}(\det A^{-} )^{1/4}e^{-\tfrac{1}{2\hbar }A^{-1}x\cdot x}\phi _{W^{\prime }Y^{\prime }}(p) \end{aligned}$$

(formula (82)) and the inequalities (120) are satisfied since we have

$$\begin{aligned} A(A+YA^{-1}Y)^{-1}\le I_{n\times n}~. \end{aligned}$$

A similar argument allows to to study the general case $AB\le I_{n\times n}$ using Theorem 12. Hardy’s uncertainty principle thus appears as being a particular case of the reconstruction theorems we have proven, and which are themselves based on the notion of quantum polar duality.

### Donoho and Stark’s Uncertainty Principle

As we mentioned in the introduction, Hilgevoord and Uffink emphasized in [30, 31] that standard deviations only give adequate measurements of the spread for Gaussian states. A good candidate for a more general theory of indeterminacy is to define an uncertainty principle using the notion of concentration of a state. It turns out that Donoho and Stark [22] have proven a concentration result for a function and its Fourier transform which can be viewed in a sense as a variant of Hardy’s uncertainty principle; as we will see it can also be interpreted in terms of quantum polar duality and is related to the Mahler volume. Let $X\subset {\mathbb {R}}_{x}^{n}$ be a measurable set and let ${\overline{X}}={\mathbb {R}}_{x}^{n}\setminus X$ be its complement (convexity is not assumed here). We will say that a function $\psi \in L^{2}({\mathbb {R}}^{n})$ is $\varepsilon$-concentrated on *X* if we have

121 $$\begin{aligned} \left( \int _{{\overline{X}}}|\psi (x)|^{2}dx\right) ^{1/2}\le \varepsilon ||\psi ||_{L^{2}}~. \end{aligned}$$

If $||\psi ||_{L^{2}}=1$, which we assume from now on, this is equivalent to the inequality

122 $$\begin{aligned} \int _{{\overline{X}}}|\psi (x)|^{2}dx\le \varepsilon ^{2}~. \end{aligned}$$

The Donoho–Stark uncertainty principle says that if the normalized function $\psi \in L^{2}({\mathbb {R}}_{x}^{n})$ is $\varepsilon _{X}$-concentrated on *X* and its Fourier transform ${\widehat{\psi }}$ is $\varepsilon _{P}$-concentrated of *P*, that is

123 $$\begin{aligned} \int _{{\overline{X}}}|\psi (x)|^{2}dx\le \varepsilon _{X}^{2}{ \ },\,\int _{{\overline{P}}}|{\widehat{\psi }}(p)|^{2}dp\le \varepsilon _{P}^{2} \end{aligned}$$

then we must have

124 $$\begin{aligned} |X|~|P|\ge (2\pi \hbar )^{n}(1-\varepsilon _{X}-\varepsilon _{P})^{2} \end{aligned}$$

for $\varepsilon _{X}+\varepsilon _{P}<1$. Taking $P=X^{\hbar }$ this shows in particular that the Mahler volume of *X* satisfies

$$\begin{aligned} \upsilon (X)\ge (2\pi \hbar )^{n}(1-\varepsilon _{X}-\varepsilon _{X^{\hbar }} )^{2}~. \end{aligned}$$

Let us apply the estimate above to the dual pair $(X,X^{\hbar })$ of centrally symmetric convex bodies. We have the following remarkable result relating the Donoho–Stark UP and the Mahler volume:

#### Theorem 14

Let *X* be a symmetric convex measurable body in ${\mathbb {R}}_{x}^{n}$ and $\psi \in L^{2}({\mathbb {R}}_{x}^{n})$, $||\psi ||_{L^{2}}=1$. Assume that $\psi$ is $\varepsilon _{X}$-concentrated in *X* and ${\widehat{\psi }}$ is $\varepsilon _{X^{\hbar }}$-concentated in $X^{\hbar }$ with $\varepsilon _{X}+\varepsilon _{X^{\hbar }}\le 1$. Then we must have

125 $$\begin{aligned} 1\ge \varepsilon _{X}+\varepsilon _{X^{\hbar }}\ge 1-\frac{1}{2^{n/2}\Gamma (\frac{n}{2}+1)} \end{aligned}$$

that is $\varepsilon _{X}+\varepsilon _{X^{\hbar }}\rightarrow 1$ as $n\rightarrow \infty$.

#### Proof

Combining the Blaschke–Santaló estimate (108) for the Mahler volume $\upsilon (X)=|X|~|X^{\hbar }|$ and the Donoho–Stark inequality (124) we get

$$\begin{aligned} \frac{(\pi \hbar )^{n}}{\Gamma (\frac{n}{2}+1)^{2}}\ge (2\pi \hbar )^{n} (1-\varepsilon _{X}-\varepsilon _{X^{\hbar }})^{2} \end{aligned}$$

and hence

$$\begin{aligned} 0\le 1-\varepsilon _{X}-\varepsilon _{X^{\hbar }}\le \frac{1}{2^{n/2}\Gamma (\frac{n}{2}+1)} \end{aligned}$$

which is (125). $$\square $$

If the Mahler volume of *X* satisfies the equality

$$\begin{aligned} \upsilon (X)=(2\pi \hbar )^{n}(1-\varepsilon _{X}-\varepsilon _{X^{\hbar }})^{2} \end{aligned}$$

then we must have

126 $$\begin{aligned} 1-\frac{1}{2^{n/2}\Gamma (\frac{n}{2}+1)}\le \varepsilon _{X}+\varepsilon _{X^{\hbar }}\le 1-\frac{1}{8^{n/2}n!^{1/2}}~. \end{aligned}$$

This follows from the estimate (113) for the Mahler volume.

These estimates show that when the number of degrees of freedom *n* is large, the sum $\varepsilon _{X}+\varepsilon _{X^{\hbar }}$ of the concentrations of a wavefunction and of its Fourier transform is practically equal to one. If the Mahler conjecture is true, then (126) may be replaced with

127 $$\begin{aligned} 1-\frac{1}{2^{n/2}\Gamma (\frac{n}{2}+1)}\le \varepsilon _{X}+\varepsilon _{X^{\hbar }}\le 1-\frac{2}{(2\pi )^{n/2}(n!)^{1/2}}~. \end{aligned}$$

For example, if $n=6$ (which corresponds to a system of two particles moving in physical space) we will have $0.979<\varepsilon _{X}+\varepsilon _{X^{\hbar } }<0.999$.
